# The impact and value of the Parkinson’s nurse specialist to people with Parkinson’s and their care partners: a grounded theory qualitative study

**DOI:** 10.1186/s12912-024-02441-7

**Published:** 2024-10-28

**Authors:** Kathryn Mcewan, Amanda Clarke, Sonia Dalkin, Annette Hand

**Affiliations:** 1 University of Northumbria at Newcastle, Newcastle upon Tyne, England; 2 Newcastle Hospitals NHS Foundation Trust, Newcastle upon Tyne, England

**Keywords:** Parkinson’s disease, Specialist nursing, Person-Centred Care, Compassion, Care partners, Long-term conditions, Personalised care, Nurse prescribing, Non-medical prescribing, Concordance.

## Abstract

**Background:**

Where available, Parkinson’s Nurse Specialists (PNS) provide a range of care, support, guidance, and advocacy for people with Parkinson’s (PwP), and, where appropriate, their care partners (CP). Parkinson’s is a complex and progressive condition. Consequently, evaluating health outcomes is not a reliable method to understand the value and impact of PNS. Previous research has identified PNS can improve the subjective well-being of PwP in the community, also that barriers to care include heavy caseloads and a lack of time. Yet little is known about the value of the role of the PNS, particularly about the impact of pharmacological management and review. This research aims to close this research gap by providing explanatory theories of the impact and value of PNS to PwP, their CP, and other professionals.

**Methods:**

A social constructivist grounded theory approach was used. Semi-structured interviews were conducted with three groups, PNS, PwP, and CP. Interviews were analysed using NVivo for coding and categorising and Word for memo-writing. Data was analysed inductively and iteratively to identify contexts, social processes, actions, and behaviours, before final emergent theories were identified.

**Results:**

46 semi-structured interviews (PNS 18, PwP 19, CP 9) led to four data categories and 13 sub-categories that delineated PNS value. (1) Expert Counsel; provision of emotional support, education, and lifestyle guidance; CP inclusion; provision from diagnosis; and across all stages of Parkinson’s. (2) Conduit of Care; signposting, referral, and connection to PwP, CP, others; PNS barriers and facilitators; (3) Team/Partnership; continuity and partnership, ‘working together’; (4) Pharmacological Support, PNS prescribing; concordance; speed of treatment. Where PNS were accessible they could offer personalised support and partnership, so providing person-centred care that improves health and well-being.

**Conclusion:**

Where a PNS is accessible due to service availability and manageable caseloads, to provide person-centred care, they deliver several benefits to PwP and CP which improve health and perceived well-being. Where PNS are not available, PwP and CP often struggle to manage their Parkinson’s with negative impacts on health and well-being.

**Supplementary Information:**

The online version contains supplementary material available at 10.1186/s12912-024-02441-7.

## Background

Parkinson’s Disease (PD) is the second most common neurodegenerative condition in the world, affecting over 6 million people [1]. For reasons that are yet to be fully understood, the incidence and prevalence of PD has risen rapidly over the past two decades, and numbers continue to rise [2]. PD is a complex condition, often resulting in motor impairments (e.g. movement and mobility problems) and non-motor symptoms (e.g. sleep problems, cognitive impairment, depression, and constipation) [3]. Those supporting people with Parkinson’s (PwP) often take a significant role in their care and health related quality of life has been found to be poor in PwP and their care partners (CP), being associated with markers of advancing PD severity, lower physical activity, higher depression severity and increased non-motor symptoms, which affects their everyday life and the ability to manage and cope with PD [4].

Currently there is no proven neuroprotective or disease modifying treatment available for PD and treatment remains symptomatic. The management of Parkinson’s is complicated due to the progressive nature of the disease, individual patient heterogeneity, and the wide range of range of both motor (e.g., stiffness and slowness) and non-motor (e.g., anxiety, depression and sleep disturbance) signs and symptoms that will increasingly impact on daily activities over time. The motor, and some non-motor symptoms (e.g. anxiety and depression) can be treated with a range of dopaminergic therapies to reduce the impact of symptoms on quality of life however, other non-motor symptoms (e.g. psychosis and orthostatic hypotension) are often worsened by treatment. Even when oral dopaminergic treatments are optimally managed PwP can continue to have debilitating response fluctuations and dyskinesias [2] and side effects such as nausea, orthostatic hypotension and impulse control disorder are frequently observed [5]. Due to the complex nature of pharmacological therapy in Parkinson’s it is recommended that PwP should have regular access to specialist clinical monitoring and medicines adjustment, and in the UK, this may be provided by a Parkinson’s Nurse Specialist (PNS).

The United Kingdom (UK) National Institute for Health and Care Excellence (NICE) guideline on PD [6] recommends that PwP should have regular access to: clinical monitoring and medication adjustment; a continuing point of contact for support, including home visits when appropriate; and reliable information about clinical and social matters of concern. The PD NICE [6] guideline suggests these resources can be provided by a PNS, a role that was originally created in 1998 as a response to recommendations from previous research [7]. There are now over 500 specialist nurses working in the field of Parkinson’s in the UK. Other countries, including Australia, Ireland, and the Netherlands, also have PNS roles, yet there is little published evidence on their effectiveness.

A recent systematic review of the literature on PD [8], highlighted the value of PNS’s delivering personalised care to PwP. Yet, this review further demonstrates how few evaluation studies have been undertaken in the UK in the last 20 years. We seek to fill this research gap with this new evaluation evidence. Specifically, we draw attention to the inclusion of pharmacological management by PNS in the UK, many PNS in this study were a qualified non-medical prescriber (NMP), a unique position internationally. The positive impact of this prescriber status is set out over the findings and discussion and provide useful evidence for this additional responsibility of the PNS to an international readership.

The PNS assists with ongoing management and follow-up through medication review; clinical leadership; help with post-diagnostic counselling; education support and advice for PwP, care partners and other staff; signposting to other services; and case management [9]. Many PNS run clinics, undertake home visits, refer on and co-ordinate care packages [10]. They are usually the first point of contact for PwP, ensuring fast access to specialist care, whilst relieving pressure on neurologist/geriatricians with a special interest in PD [9]. To support the PNS within their role, the Parkinson’s Competency document [11], describes the knowledge and skills required by all PNS to manage the care of PwP across healthcare settings. Parkinson’s UK provides an induction training programme for new PNSs and a learning pathway to signpost and suggest areas of further education/development and to support newly appointed PNSs and more experienced PNSs.

While little research about the PNS role has been undertaken, previous research demonstrated that PwP living in the community and supported by a PNS had improved subjective wellbeing at no extra cost, compared with those who were supported by General Practitioners (GPs) [12]. Furthermore, compared with neurologists, PNS provided longer consultations and paid more attention to patients’ concerns [13]. No differences have been found between the two job roles in terms of health outcomes for PwP, although there is some benefit to the two professions collaborating [13]. When PNS were surveyed to examine job specification, perceptions of service delivery and views about assistance, the major barriers to service delivery were identified as heavy case load, lack of time, and lack of clerical help. Another evaluation of the PNS role examined the perceived effectiveness, acceptability, and efficacy among PwP, their care partners, and the multi-professional team (MPT) [14]. One key finding was the perceived value of the PNS in hospital and community settings, yet further clarification of the PNS role in these settings was required. A more recent Swedish study demonstrated the PNS role in providing tailored and competent care to alleviate the impact of PD on daily life if they had the practical skills, theoretical knowledge of PD, and the ability to provide emotional support [15]. One specific further competency of PNS is they can hold a non-medical prescribing (NMP) qualification; worldwide, NMP is expanding yet UK nurses have been prescribing the longest.

PD is a progressive condition, with all PwP showing levels of deterioration over time; therefore, using health outcomes is not a reliable or effective way of evaluating the value of the PNS. To overcome these challenges and better demonstrate the value of PNS to PwP, this article describes the first study incorporating the experiences, perspectives, behaviours, and actions of the PNS, PwP and their CP. We bring forward new evidence on the impact and value of the role of the PNS, specifically including the delivery of personalised care, also known as person-centred care (PCC) [16]. An ever-evolving concept over recent decades, PCC has been increasingly central to health care practice [17]. PCC encourages the individualisation and personalisation of care, working in partnership with service users, so doing *with* rather than doing *to* or *for*, practitioners are encouraged to centre compassion, engagement, and communication [17]. Our findings bring new evidence to how these appear in practice and the impact this has on patients, caregivers, and nurses.

## Methods

The purpose of this study was to interrogate the differing impacts and values of PNSs on PwP and their CP to build towards a greater impact assessment of the role; we aimed to achieve this through the production of explanatory theories with aligned service recommendations. The following research questions were addressed:

1. What are the differing impacts and recognised value(s) of specialist nursing on PwP and their CP?
2. What are the barriers and facilitators for PwP and their CP to receiving care from a PNS?

The study was developed and overseen by an expert panel including: the research team, Parkinson’s Disease Nurse Specialist Association representatives, nurse leads within the Parkinson’s Excellence Network, the Parkinson’s UK Service Development and Improvement Lead, and a Patient and Public Involvement (PPI) group.

Our PPI group, which included PwP and CP, contributed to the design of the study, promotion of the surveys, design of the surveys’ interview guides, validation of findings, and dissemination of early findings.

### Design

The study was guided by a social constructivist grounded theory methodology and incorporated an inductive approach [18–20], . Given the lack of previous research, and the extensive differences in services and experiences across the UK, adopting this study design allowed for the recognition of the fluidity of realities and to properly represent the participants voice within [21].

The study commenced in April 2019 and closed April 2021. Initial stages included stakeholder information gathering and literature scoping to build up early theorising which could be tested and reviewed through data collection to produce final theories grounded in the data [19].

### Study setting and recruitment

Participants were PNS, PwP, and the CP of PwP. They were recruited across the UK (Northern Ireland, Scotland, England and Wales). Potential participants were invited to answer some ‘about you’ questions on a well-promoted online portal and indicate whether they could be contacted for interview. PwP and CP could participate in the study independently of each other. While this allowed for a range of populations and settings, and so insights, we recognise our sample will still be inherently biased towards those who were interested and able to respond to an online request for participation.

#### Inclusion criteria:

1. Specialist nurses identifying themselves as working with PwP as a major part of their role.
2. People with Parkinson’s, with a confirmed diagnosis of PD, cognitively able to participate.
3. Informal care partners of a person with (diagnosed) Parkinson’s.

The research team recruited PNS, PwP, and CP who met the inclusion criteria through an online portal which was promoted through:

- Healthcare services: Parkinson’s specialist services, PNS, The National Institute for Health Research Clinical Regional Networks.
- Community organisations and groups: Parkinson’s UK, local support groups, Parkinson’s disease Nurse Specialist Association.
- Adverts through social media (Facebook/Twitter), PPI networks, Parkinson’s UK webinars.

### Sampling and data collection

By maintaining a small database, on Microsoft Excel, of ‘about you’ responses from the online portal, we were able to undertake sequential sampling. Initially, utilising selective sampling, then moving through iterations of concurrent analysis, we could follow the data and apply theoretical sampling to test arising patterns [22].

Preliminary selective sampling was undertaken from the database to include a range of circumstances and experiences. For example, for nurse participants we interviewed across a range of professional bands, with bands 5–8 included in the data. UK nursing bands represent different levels of education, experience, and responsibilities; band 5 is an entry level position and band 9 is the most senior position. While we had close numbers of self-reported women and men participants in PwP interviews (10 women and 9 men), there was gender disparity in our nursing sample (16 women and 2 men), and in our CP sample (6 women and 3 men). This difference, with significantly more women than men responding and participating, reflects similar gender disparity in the fields of nursing and caring in the UK [23] and in informal caregiving [24]. We also used this approach to ensure we interviewed nurses from a mixture of hospital and community settings, from urban and rural locales, and who worked alongside different consultants (e.g. Neurologist and Elderly Care Physicians). To better understand the range of experiences of PwP and CPs, we also selectively sampled from those who had current access to a PNS, those who had previously had access but had lost this, and those who had never had access. Similarly, we sampled from a range where online respondents had indicated they had a positive, neutral, or negative experience with services to avoid overly positive bias. See Table 1 for more details of the sample.

Individual semi-structured interviews were conducted with all three participant groups online on Microsoft Teams. These followed a topic guide co-developed by the research team and PPI group using early coding and categorising, in line with on-going emergent theory development [25] (see Supplementary File 5 for all interview topic guides). Interviews were undertaken by one researcher (KM) between September 2020 and January 2021, lasting between 35 and 90 min (average 55 min), were digitally recorded, and transcribed verbatim. No participants withdrew from the study. Iterative analysis and recruitment continued until data saturation, which we refer to as theoretical saturation, was agreed to be reached during the team analysis meetings [25, 26].

### Data analysis

A total of 46 semi-structured interviews were conducted (PNS 18, PwP 19, CP 9) (see Table 1 Interview Participants). Pseudonyms were attributed to participants and any identifying markers were removed from transcripts before they were entered into NVivo 12 Pro (NVivo qualitative data analysis software; QSR International Pty Ltd. Version 12, 2018) for management of data and the analysis process. Microsoft Word and Teams were used for memo-writing and theory building.

**Table 1:** All interview participants

	PNS (18 Interviews)	PwP (19 Interviews)	CP (9 Interviews)
Country (UK)	1x NI 2x Wales 2x Scotland 13x England (11 regions)	17x England (15 diff regions) 2x NI	9x England (9 areas)
Skills and Competencies	10x prescribers 8x non-prescribers		
Experience Range	Qualified 1985–2015		
Gender	16x Women 2x Men	10x Women 9x Men	6x Women 3x Men
Age Range		1 × 35–44 1 × 45–54 5 × 55–64 7 × 65–74 3 × 75–84 2x no info	1 × 35–44 1 × 45–54 2 × 55–64 3 × 65–74 1 × 75+ 1x no info
Time Since Diagnosis		11x < 6yrs 6 × 8-13yrs	2x < 5yrs 1 × 5-10yrs 4 × 10-15yrs 1 × 15-20yrs 1x uncertain
Relationship with PwP			5x spouse 4x daughter/son
Years as Carer			1 × 3yrs 3 × 10yrs 1 × 13yrs 1 × 15yrs 1 × 20yrs 1 × 30yrs 1x uncertain

Principles of a constructivist grounded theory approach to analysis were used. An iterative process was undertaken to identify arising patterns of incidences, exceptions, (re)actions, and experiences within a range of contexts, from the interview transcripts [18–20]. Maintaining this iterative approach allowed us to be dynamic in the interviews, by which we could use theoretical sampling to include different populations as we progressed, and adding notes to the interview topic guides to query arising patterns, codes, and categories [22].

Initial coding began alongside data collection, followed by focused, and then theoretical coding [25]. The researchers (KM, AH) worked jointly and independently, to provide checks and balance on each other’s coding to ameliorate bias, challenge pre-conceptions, and improve reflexivity [19]. Similarly, where potential contradictions appeared, this was discussed by the researchers and abduction applied to make connections [27]. Codes were grouped into data categories and sub-categories, identified, and organised through their apparent explanatory potential using constant comparison of data [19]. Memo-writing was conducted through researcher and wider research team discussions of data in regular online meetings, where we deliberated the relationships between arising concepts and categories and encouraging challenges and confirmations [25].

Rather than coding saturation, we sought meaning saturation. We continued to collect data until we could understand differences, perspectives, contexts, and outcomes for the study population [26]. Due to this approach, we were able to agree theoretical saturation, wherein between the codes, categories and theorising, and our differing levels of knowledge, we could agree that new properties of categories were no longer being discovered, variability was accounted for, and relationships had been tested and validated [26].

At this point, emergent final explanatory theories were produced, and subsequent recommendations made (See Supplementary File 6 for a full list of recommendations). A working model of how the interview questions fed through the data collection into the analysis and into recommendations is provided in the supplementary files (Supplementary File 1). These findings are reported according to the reporting and evaluation guidelines for grounded theory research studies (GUREGT) developed by Berthelsen et al. [25] (see Supplementary File 4 GUREGT checklist).

## Results

Four data categories, each with several sub-categories, were identified (see Table 2: Data Categories and Sub-Categories). Generated following extensive coding practices, these different categories depict the role of the PNS and the differing impacts and values of the role, alongside the social process, behaviours, and actions as PwP and CP access and engage with them (or not). For an example of how qualitative data was coded into (sub)categories see supplementary files (Supplementary File 2 and Supplementary File 3).

**Table 2:** Data categories and sub-categories

Category No.	Category Name	Sub-Categories (1)	Sub-Categories (2)
1	Expert Counsel	Emotional/Educational/Lifestyle Advice/Support/Guidance; CP Inclusion	From diagnosis; Across all Parkinson’s stages
2	Conduit of Care	To PwP; To CP; To HSCPs	Barriers and Facilitators to PNS/Care/Support
3	Team/Partnership	Continuity and Partnership	Working Together
4	Pharmacological Support	PNS prescribing	Concordance; Speed of Treatment

**Fig. 1:**
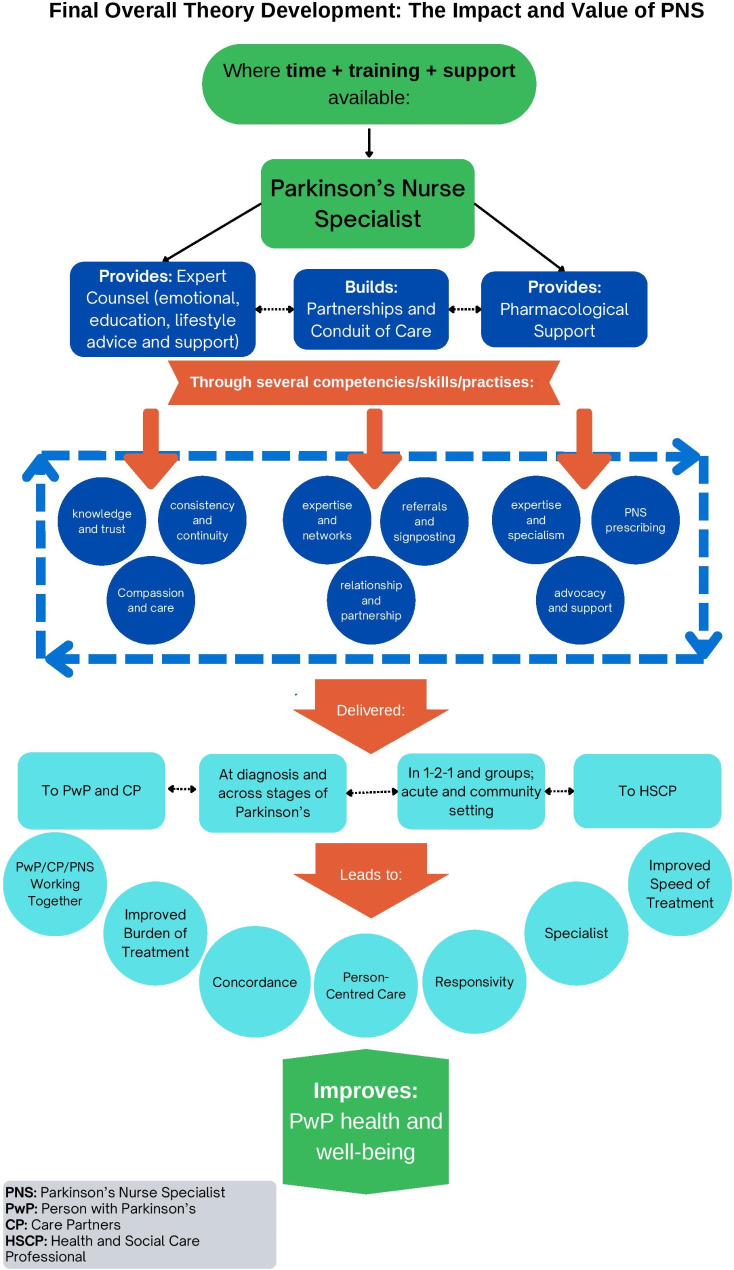
The value and impact of the parkinson’s nurse specialist

Data from categories: 1 Expert Counsel, 2 Conduit of Care, 3 Team/Partnership and 4 Pharmacological Support is outlined below. Each sub-category ends with the emergent grounded theory (GT) for that area. All the emergent theories are then drawn together into a visual overview of the overall findings (see Fig. 1: The Value and Impact of the PNS) and a final overall grounded theory is stated.

### Data category 1: expert counsel

Expert counsel encompasses subcategories: (i) Emotional support; (ii) Education on Parkinson’s; and (iii) Lifestyle advice and guidance. This expertise needs to be provided in, and over, time with a PwP and, where appropriate also with the CP. That is to say, when ‘expert counsel’ was provided by a PNS to a PwP, preferably from diagnosis, and continuously across the stages of the condition, this ultimately led to improved self-management and/or improved perceived well-being. Where this was extended to CP’s, there was an additional benefit to the flow of information between the PwP and the PNS, and an improvement in perceived well-being for the CP.

**Sub-category: emotional support**

A distinctive characteristic of a PNS consisted of their ability to mobilise the key resources of empathy; understanding, time, and emotional support in a meaningful way that is specific to the needs of the PwP and their family environment. For PwP, this was distinctly different to other forms of support and positively impacted their perceived quality of life, partly because meaningful conversations came about in flexible, informal ways. For example, arising from a conversation rather than an assessment, and are focused on ‘the person’.

*It is more than just a GP*,* I don’t want to do them down*,* but [GP appointment] is less intimate*,* [PNS appointment is] half an hour or an hour long and she gets to know me much more than a superficial level; work and daily managing and how I am doing physically and mentally. And the emotional support*,* she is more in tune than I am sometimes*. (PwP14)

Additionally, at the heart of a meaningful conversation is the ability of the PNS to listen and give people time to talk and express their feelings.

*Many relatives*,* once I have spoken to them in any appointment*,* they say you don’t realise how much reassurance and comfort you have given to [PwP]*,* they feel so much better when they’ve seen you. The reassurance they are doing well and the hope we give them.* (PNS1.4)

Providing this responsively provided a sense of companionship.

*[…] It was a bit like a walking stick that comes along beside you and not all the time just in the walk when you need the stick.* (PwP18)

#### Emotional support grounded theory (GT)

When PNS’s have time available (within an appointment and in caseload to meet regularly) they can utilise their communication skills to provide individualised and authentic emotional support to PwP and their CP which improves perceived well-being.

**Sub-category: education and lifestyle advice**

Education was understood to be the provision of individualised pertinent information about Parkinson’s from a PNS, via one-to-one contact or groups. Such education was seen as a sharing of expertise that supported PwP to better understand their condition. Where this was missing, it was more difficult to manage changes.

*You need to have the education to implement the lifestyle choices […]. You need to understand what it means to have issues with swallow and how that works and why that is happening and what the risks are […] but I think you need the education to understand about the disease to […] be able to implement the practical things on a day-to-day level.* (CP7)

Where the PNS was unavailable to provide education, PwP and CPs were left to seek their education elsewhere. Although this could be from reliable sources, (often identified as Parkinson’s UK networks, groups, phone line, or website resources), many were left scouring all resources online without the understanding to sift these for appropriateness and so act on the information accordingly. Or they were reliant on consultants or GPs who might not have the time or specialised information for appropriate education.

*[With no PNS we would find] research taking place in other countries and there is lots of holistic stuff and it sorts of muddy the waters*,* especially for mum who [would] read things and would act on it and then it wouldn’t work*,* and it would be so disheartening.* (CP1)

Lifestyle advice was indicated to be a more informal provision of knowledge, this was understood to be best provided by a PNS; they were able to provide individual actionable guidance through their established relationships with PwP.

*When I see a patient*,* we go through everything quite holistically I try and de-medicalise it quite a bit as they get enough of that.* (PNS1.12)

#### Education and lifestyle GT

When PNS’s have time available (within an appointment and an appropriate caseload to meet regularly) they can share personalised education and lifestyle advice that provides PwP’s confidence and reassurance, this empowers them to manage their Parkinson’s, improving their health and well-being.

**Sub-category: CP inclusion**

The role of the PNS is to the support the PwP to manage their symptoms, provide information and guidance, and enable an individual to self-manage their condition (where appropriate). As Parkinson’s progresses, due to its disabling nature, many PwP will require support from CP (informal or formal) to assist with day-to-day activities to enable them to remain living within their own home.

*When you say Parkinson’s nurse specialist you are thinking of a one-to-one intervention*,* but it is not*,* it is the whole family […]*,* because everyone is affected by Parkinson’s.* (PNS1)

Where this was appropriate, the inclusion of CP’s by the PNS in their continuous provision of ‘expert counsel’ had a positive impact, reducing experiences of strain and burden, and improving the relationship between the PwP and the CP.

*I didn’t expect to get anything for me*,* but I know I benefited from [PNS] […] helping me helped mum in the background as I had a way to get support […] so I didn’t stress about it in the same way as I had been able to unload.* (CP2)

There were challenges for the PNS, some noted the difficulties of negotiating inter-familial relationships and maintaining balance, which required careful management. Yet, including CP’s improved information flow. PNS’s explained CPs could provide additional knowledge about the day-to-day symptoms and experiences of the PwP. While the ability to ‘offload’ about their experiences could reduce strain on CPs. PNS shared that CPs have a significant input into medication management at home.

*Sometimes that advice can prevent hospital admissions.* (PNS1.10)

Topics which PNS and CPs discussed included managing issues with impulse control and associated household debts, suicidal ideation, cognitive impairment, managing changes in household and relationship roles, adapting to different stages and transitions, medication, and titration. PNS provided reassurance, advice, and support.

*When they move to complex and palliative stages the emotional support becomes more important […] to talk through what they are feeling and their emotions […] is so important. I was up all night with my son sick and I went into work*,* and I thought*,* gosh*,* this is what our carers do all the time […]*,* our link with them is equally important as with the patient.* (PNS1.6)

#### CP inclusion GT

CPs provide on-going and increasing modes of care to PwP. Where they are included in PNSs ‘expert counsel’, they can help ensure personalised care, better supporting the PwP, and receive advice, support and guidance which reduces caregiver strain.

**Sub-category: diagnosis**

For some people, diagnosis can be a stressful time. Getting the right information and support from the beginning is crucial. When a PNS provided ‘expert counsel’ to the PwP early in the disease process, this led to reassurance, resulting in improved well-being.

*Your world falls apart when you get your diagnosis*,* and she helped me put it in perspective.* (PwP2)

PNSs described supporting PwP to make sense of the diagnosis. By providing ‘expert counsel’ early, the PNS could manage expectations, setting the PwP on the ‘right road’ to understand their condition and their new future.

*When we first got the diagnosis*,* the Parkinson’s nurse was brilliant*,* and I couldn’t rate her more highly. She was round to the house quite often as (PwP) was still working then so trying to fit in appointments was difficult and she would come when she knew he was off duty.* (CP5)

Where the PNS was unavailable at diagnosis, PwP could be left without knowledge, support, and contacts, with negative implications for their well-being.

*The day I was told*,* I went to an appointment at the hospital and they say off you go […] without any tablet or treatment and he said I will see you in a year […] and I eventually went back to GP as I had terrible aches and pains and he sent me to an orthopaedic guy and waited for 3 months to see him […] and he said I am not a Parkinson’s specialist but you are suffering with Parkinson’s and I will refer you back to the consultant but it took about 4–5 months.* (PwP13)

#### Diagnosis GT

Diagnosis can be a stressful time, PwP require access to a PNS and their ‘expert counsel’ from diagnosis to improve their confidence and ability to manage their Parkinson’s.

### Data category 2: conduit of care

PNSs provided a valuable conduit role to PwP, supporting their connections and engagement with other appropriate NHS, Local Authority, and third sector services. They also provided a comprehensive conduit role for CPs, as an extension of care to the PwP, working with other agencies and organisations to increase CP’s knowledge and care of PwP outside of their immediate contacts.

**Sub-category: conduit of care for PwP and CP**

Where the PNS had time available to build relationships with PwP, they could act as a ‘conduit of care’. This manifested in multiple ways, from information sharing and signposting, to referral, to advocating on behalf of PwP, in direct and indirect ways. These activities supported the co-ordination of care around the PwP, ensuring they received appropriate and timely treatment and were signposted to additional useful support as required.

PNSs and PwP often described attending community groups where support could be accessed by those potentially unable to (regularly) attend clinic, extending the boundaries of care. All participant groups explained being able to access symptom management guidance, general advice, and well-being support through this avenue. PwP and CPs described how these groups provided opportunities for others to ask the questions they were fearful to ask or unaware of themselves.

*At [local Parkinson UK group] sessions the nurse is very knowledgeable*,* and people always say “I didn’t know that” […] when [PNS attends] everybody seems to benefit as they pick up things they didn’t know and also they find that [PNS] will stay on after the meeting and just catch up so people can put a name to a face.* (PwP18)

PNSs explained how they would act as a conduit into a Parkinson’s service, ensuring the inclusion of a GP to provide a formal referral. Once in this specialist service, the PNS acts again as a conduit to PwPs; providing referrals, signposting, and advice for any PwP to receive further appropriate care.

*There is one role [where] you are a coordinator*,* and another role is detective as you have to get to the root cause of the problems*,* you have to look at all the rationale*,* and once you have done that you refer them onto the most appropriate person to deal with that.* (PNS1.8)

Another way of describing this was working as a ‘hub and spoke’ approach:

*So*,* psychiatry*,* GP’s*,* rehab teams in the community […] they all kind of come in and […] when you look at the spokes for the patient*,* we are the centre of the wheel*,* and that can be really useful for patients having one point of contact in that regard.* (PNS1.1)

The PNS had knowledge of, and contact with, multiple disciplines and services to obtain the comprehensive signposting and information sharing PwP required, including: advocating for, or providing contacts and support to self-advocate with, GPs, social care, physiotherapy, speech and language services, mental health services, occupational therapy, hospices, rapid response, district nurses, dieticians, crisis management, bladder, and pharmacies.

Subsequent engagement was improved when the PNS acted as a ‘conduit of care’.

*The classic example you hear [is] “I don’t want to bother them”*,* because they see and view their own problem as trivial whereas it is not. So signposting is not just telling people they can do things it is giving them permission to go and access that information and access that organisation and that they are not wasting anyone’s time.* (PNS1.1)

Difficult conversations could be had which improved opportunity to share information.

*[nurse] knows as a man I am very independent and if she sees me struggle [she] always asks*,* how is [wife] feeling? I haven’t told anyone till the other day*,* but I do suffer from depression*,* and I haven’t even told me wife*,* but [nurse] is now going to give me help for someone to talk to.* (PwP19)

*[PwP] had been on a dopamine agonist and she had a lot of nice expensive jewellery on and […] we talked about impulsive behaviours and she burst out crying and I realised she was in a huge amount of debt and that was making her symptoms worse and that was making the consultant put the dose up*,* so we got involved and the Age UK worker was able to go in and actually send things back and get debt written off and now she has no compulsivity and no debt*. (PNS1.6)

There were barriers to PNS’ attempts to provide this conduit role. Primarily, that the service referred to must be accessible and available. Mental health service availability was a particular problem.

*Here*,* we can’t make referrals to memory services as they are medic to medic*,* and you have to ask the consultant and they will say ask the GP and you get stuck in the middle.* (PNS1.15)

Examples of barriers to taking-up referrals included when a PwP was offered services outside their geographical location (not available locally), or they did not want to see others in the later stages of Parkinson’s:

*I don’t particularly want to [get involved or get more support]*.* I am not in denial*,* but I do want to carry on the rest of my life best I can with Parkinson’s*,* and I don’t want to go to a meeting where people are suffering badly.* (PwP1)

Where there was a continuous relationship of care, the PNSs could encourage engagement post-referral.

*I do follow through [on referrals] but […] my interest tends to wane […] unless I am pushed by [PNS] I do back off. […] I do tell people I am lazy*,* but I don’t make excuses I […] need to be reminded and nudged.* (PwP10)

#### Conduit of care to PwP and CP GT

PNSs provide a ‘conduit of care’ to PwP and their CP, their extensive knowledge of not only Parkinson’s Disease, but also of the individual PwP, their networks and relationships with other HSCPs and agencies, allow them to facilitate appropriate referrals and signposting, and the encouragement to engage.

**Sub-category: 'conduit of care’ for health and social care professionals**

The practice of (in)formal HSCP education was varied yet consistently present. Formal practice included pre-arranged presentations or lectures, provided in-person or online. These were viewed as an important aspect of the PNS role, albeit problematic due to low availability of staff time, for both the PNS and other HSCPs they sought to support. Learning materials for these sessions were produced and provided predominantly by the PNSs.

Informal education was also described as a regular activity, occurring ad hoc on hospital wards, in care homes, in community settings, and to multiple, different clinical staff and professions; including, GPs, dentists, pharmacists, physiotherapists, occupational therapists, care/nursing home staff, community/district nurses, and hospital medical staff/nurses.

*Yes*,* normally we do a lot of teaching. Since Parkinson UK pulled money for educating that has come back on us*,* and it takes timeout during the day*,* but it works out in the end as [HSCPs] don’t need to call as much.* (PNS1.11)

PNSs explained why training to HSCPs was so valuable, including that this (in)formal education could improve a hospital experience for PwPs.

*I have a formal education plan every year and all members of staff in the acute trust are enabled to attend and I do a lot of information education on the wards and in the emergency dept with junior doctors and registrars so there is a lot of formal and informal education and I do that to improve patients experience when they come into hospital.* (PNS1.10)

As a more ‘hidden’ part of the PNS service this education was often the first to be relinquished under pressure and PNSs felt strongly this had a negative impact on PwPs and other services. Nonetheless, for some PNS, time constraints meant HSCP education could not be maintained.

*It’s hard getting the time now as I only work part time no one helps you prepare for your sessions […] because of time we just can’t afford to do it.* (PNS1.5)

#### Conduit of care to HSCP GT

PNSs provide a ‘conduit of care’ to HSCPs through extensive (in)formal education to ensure better care for PwPs as they encounter them.

### Data category 3: team/partnership

PNSs, PwPs, and CPs described working in a team, or in partnership, with each other to live well with Parkinson’s. The PNS ‘got it’ and ‘got things done’.

Where a sense of partnership had been developed, the PNS was the main pillar of support.

*If I had to pick one support*,* I would go for the nurse they have all been approachable and have an extensive knowledge and have an insight into the practicalities of living day to day with it. So*,* I am a big fan. And the consultants are all nice and I haven’t had a bad experience*,* but the [PNS] get it. […] They understand the realities of day-to-day life with it. […] She gets things done.* (PwP11)

The PNS saw the person with Parkinson’s as much as the Parkinson’s.

*[If I could not easily access PNS] I would feel a bit isolated and as I say I feel it is too detailed a condition for a GP to know all the ins and outs and they have a general understanding but the knowledge the nurses have*,* the clinical application of the drugs and services that are available*,* they are much more in tune with what is relevant and available where the neurologist have a higher level view. They are not detached*,* and I am not doing them down the neurologists*,* but they don’t have the detailed information about the individual*,* so they don’t sometimes see the nuance.* (PwP14)

**Sub-Category: Continuity and Partnership**.

PwP clearly identified the importance of the PNS to them and the management of their condition. For many, the PNS was the first professional to contact for support, leading to positive perceived well-being.

*What patients will discuss with their consultant will be very different to what they will discuss with the nurse.* (PNS1.10)

GPs were often described by PwPs and CPs as not having the knowledge or skills to manage the condition.

*There is no way I would go anywhere near the GP as he hasn’t got a clue.* (PwP6)

*We have a lovely GP*,* but he is the first to acknowledge he is clueless about Parkinson’s. If*,* heaven forbid*,* they decided to remove the Parkinson’s Nurse he would be a disaster as he just doesn’t have the background knowledge or experience […] I think certainly having access to [nurse] has taken a lot of people from otherwise using other bits of the NHS. My sense is it is more efficient to have a specialist [nurse].* (CP9)

PwP also often expressed a preference to see the PNS rather than the consultant.

*Sometimes*,* I would go to see a different consultant […] and then you might get conflicting information and suggesting and that was a bit unsettling. […] So I stick with the nurse*,* as I see her more often.* (PwP3)

One PwP, who previously had access to a PNS, was able to reflect on the negative impact that no longer having that support meant to them.

*You could ring [nurse] up and leave a message and you knew you would get an answer and she would understand. […] when we lost [her it] was a big gap […]. It’s a waste of time going to your GP […] it would be like going round a loop*,* [whereas] if we rang the professional*,* we would have more chance of finding help […] I would explain it as essential [having nurse]. It was an overall reliable assistance and support. It was a comfort knowing she was there.* (PwP4)

Without a PNS, some felt there was no professional to support them with their condition.

*I wouldn’t go to the GP*,* they are overloaded*,* and they can’t help*,* they have no understanding really*,* so the GP is not my first or second port of call. […] There is really nobody on the professional level.* (PwP2)

Some PwP had never had access to a PNS but were still able to identify the potential benefits that a PNS could provide.

*It would be amazing to have someone who understands*,* who you could go to [with] the things you don’t want to talk to the consultant about [and] the GP doesn’t understand*,* would be massive both emotionally and physically […] we would just like to ask what do you do with this? […] but we are just left on our own and you feel like you are floundering.* (CP3)

PwPs and CPs also expressed concerns about potential lack of succession planning and/or losing access to their specialist nurse.

*She is a very experienced nurse*,* and she has gone down from full to part time and I am terrified she will retire as I think to get someone of her calibre again would be difficult. My GP is very hard to get hold of and knows less about Parkinson’s than I do. My consultant*,* like many*,* are almost impossible to access.* (PwP15)

*The caseload in the area is considerable*,* so one of things I think we need to be worried about is with more people being diagnosed and diagnosed earlier there is going to be a natural demand for access to more Parkinson’s nurse services […] and the other thing is succession planning and where is the experience that is going to replace her*. (CP9)

#### Continuity and partnership GT

Parkinson’s is a complex life-long condition where a PwP will transition over several stages, each bringing different challenges. Where a PwP and their CP can build a relationship with a PNS, they can better manage changes and feel reassured they have a compassionate companion.

**Sub-category: ‘Working Together’**

Where a PwP had a PNS available, they could work in partnership with them to manage their Parkinson’s.

*When we were making*,* […] joint decisions about the medication [nurse] would give me the information about different drugs in detail*,* so it didn’t feel imposed*,* she educated me about them and their effects and then I could make decisions.* (PwP3)

CP felt a similar sense of partnership.

*I think I would say that I work in partnership with a number of people and each of them is important […] the nurse and the [consultant] and [formal] carers as well. I know I couldn’t manage on my own.* (CP4)

Participants described the ebb and flow of requirements for contact but the importance of the overall continuity to maintain this partnership.

*We do tweak [medications] at home*,* and they know we tweak. We report back. We do work in partnership with them. [It makes a] huge difference just to know that somebody [is contactable] the thought of having to wait for an appointment when you are up many times in the night with someone who is confused would be horrendous.* (CP5)

Sometimes, where a long partnership had developed, PwPs or CPs explained it was difficult to imagine the PNS not being there.

*We absolute rely on the Parkinson’s nurse for [PwP] wellbeing and it is hard to conceive what situation we would be in if she wasn’t there […] we have the most confidence and respect in her ability and we absolutely know we can rely on her to respond in a professional and respectful way. And I know that other people have similar feelings of support and commitment to their Parkinson’s nurse.* (CP9)

PNSs identified it was important that PwPs and CPs, had a point of contact to provide continuity of care, but also to develop an open and honest relationship.

*I think it is important as well that patients have continuity so that you developed a relationship and that has to be open and honest as you might meet them at diagnosis and [be] doing end of life planning as well so has to be a confidential and respectful relationship. [PwP will say] you are the only person who will be absolutely honest with me about the challenges I am facing.* (PNS1.10)

#### ‘Working together’ GT

Where PNSs have the time and competencies to provide person-centred care, a partnership is developed between the PwP, CP, and PNS. This partnership, requiring continuity of care, allows for the exchange of pertinent information which can improve health and wellbeing outcomes.

### Data category 4: pharmacological management and support

In both the surveys and the interviews, all three participant groups (PNS, PwP, CP) identified some form of value gained where a PNS was able to undertake pharmacological management and support. Notably, it was indicated it improved access to individualised and appropriate treatment, concordance, and speed of treatment.

*If I wasn’t prescribing*,* I feel I would be working with one hand tied behind my back. (PNS1)*

The pharmacological management of Parkinson’s is challenging; becoming increasingly complex as Parkinson’s progresses and adjunct treatments are required to manage deteriorating symptoms. The accountability and responsibility associated with the role of an independent prescriber cannot be underestimated.

*I don’t think I slept for about a year; it is absolutely terrifying as a nurse to prescribe. […] you worry a lot*,* [but] it definitely made my practice stronger. (PNS2)*

PNS offered a wide range of pharmacological support, initiating treatment, titrating medications, or providing advice on medications. PNS prescribed not only Parkinson’s medications, but also other medications to treat and manage many of the non-motor symptoms that PwP may experience.

*Sometimes*,* I get consultants who feel that I should be prescribing for blood pressure and bladder and meds [that] I wouldn’t initiate without a consultant or GP as it is not in my areas of expertise*,* […] we should be working in our area of expertise and sometimes consultants do prescribe*,* and there can be contraindications and I am very aware of that.* (PNS1.9)

#### Pharmacological support GT

While not all PNS prescribe, most recognise the value of non-medical prescribing (NMP) to professionals, patients, and wider team functioning, and are keen to work towards this status. Yet NMP is a complex and responsible position which requires confidence building, specialist training, and understanding of the parameters of the role in wider teams.

**Sub-category: PNS (specialist) prescribing**

The data overwhelmingly demonstrated the value of the PNS obtaining qualification as an independent prescriber.

*I didn’t even know you could have a nurse where they didn’t prescribe. Yes*,* we are lucky in that [PNS] can and does prescribe.* (CP9)

Parkinson’s nurses were recognised as a three-fold specialist for providing pharmacological support. The PNS had expertise in Parkinson’s disease, they also had extensive knowledge of the specialist medications prescribed in the field, and finally they had an established relationship with the PwP which provided them insight into an individual’s Parkinson’s (PD can be extremely variable).

*A GP phoned me and asked me to see this particular patient as wasn’t sure drug regime was correct […] so I phoned him to give him my findings and he said*,* I will stop you there*,* I have over 300 drug information sheets in front of me*,* that’s why I send him to a specialist […] when you listen to the person with Parkinson’s […] you can key in how their drugs might work to the optimal treatment.* (PNS1)

PNSs were often undertaking advocacy on behalf of PwPs and CPs for expensive and complicated medicines to ensure appropriate treatment plans are continued by GPs.

*Sometimes we get push back from GPs. So*,* anti-sickness drugs they won’t prescribe*,* we use a lot of drugs that are off licence or off label and we get a lot of bite back from that from GPs. If we go for liquid Sinemet it is a lot more than tablets and they push back on that.* (PNS1.2)

*My responsibility is to supply the initial prescription and I will write to the GP asking them to change therapy but then I also tell the person with Parkinson’s that they need to call the surgery when they get the letter as I might supply a prescription and I see the patient and then haven’t had it continued. GPs have been known to change from one brand to another as it is cheaper*,* or they don’t understand the medication and I have had modified release being given for three times a day.* (PNS1.10)

PNS on occasion extended advocacy beyond the GP to community pharmacies to ensure treatment plans are maintained as prescribed.

*They can take [PNS] prescription to their pharmacies. And some pharmacists were confused by our purple prescriptions*,* and you can have issues like that. I had previously had GPs swop patients without their consent to cheaper forms of medication and some people can have adverse effects and*,* in that case*,* we have just asked them to be switched back […] as GPs [sometimes] go for the cheapest but that doesn’t always work out best.* (PNS1.9)

#### PNS (specialist) prescribing GT

PNS are specialists in Parkinson’s Disease, Parkinson’s medications, and an individual’s Parkinson’s (which can be extremely variable). Consequently, they can provide personalised pharmacological support with positive impacts on PwPs health and well-being.

**Sub-theme: concordance**

We found that when PNS were non-medical prescribers, PwP concordance with treatment regimens was positively impacted. This was due to the inclusion of the PwP in pharmacological discussion and review, itself a consequence of the strength of the relationship between the PwP and the PNS.

Due to the reassuring nature of the relationship between PNS and PwP, pertinent information could be shared during review which led to improvements in personalised care.

*Some of the more elderly patients they can have the consultant is God syndrome and they feel more comfortable talking to you.* (PNS1.13)

*I stopped taking the medication when I was newly diagnosed as I was very scared about the fact that I knew that it wears off and eventually it doesn’t really work […] and*,* because [PNS] wasn’t judgemental*,* I could tell him I was worried about side effects and I was trying to find out what they were and how the dose might give me side effects.* (PwP6)

The trusting relationship between PNSs and PwPs provided the latter with the confidence to ask and answer questions honestly, improving personalised care, and so, concordance, ultimately improving potential outcomes.

*You sit a patient down in clinic and you say ‘what times are you taking medication?’ and they say*,* ‘oh well I don’t always take it regularly’ and we say ‘it is so important you take it at the same time every day’ and you see a light bulb moment*,* it is not ‘I am getting worse’*,* it is ‘I am not taking medication properly’.* (PNS1.6)

*I am interested to understand what is happening and how these things work and alternatives in terms of timing and dosages and having a discussion to find the optimal fit for my situation.* (PwP14)

Where PNS had built these trust-based reciprocal relationships, they could share the reasoning behind their treatment recommendations.

*[PwP] was having shorter and longer period on and off and so [PNS] prescribed going onto another drug […] and she talked through the practicalities of that*,* such as when is best to take it*,* so meals and protein and diet related things*,* and understanding the science behind it and she can share that in everyday language.* (CP9)

This approach was often described as a contrast with that taken by some consultants.

*I have had incidences where [my] Neurologist has waved his hand and said ‘oh*,* start taking this thing’ but hasn’t been very clear with quantity or frequency or why I have been taking meds and why it is [such] level [or] tell not to take it if not providing benefit.* (PwP14)

The reassurance provided from the delivery of personalised care alongside pharmacological review was greatly appreciated, and it was missed when access to PNSs had been lost. This loss lead in some cases to reductions in concordance.

*The Parkinson’s nurse explained and put me onto a scientific paper I read that showed there was no ill effects from early taking Levodopa. And she knew that about me and understood I needed to see the paper as she understood me*,* she gave me information that was appropriate to me. […] they are powerful drugs*,* and they impact on your body*,* these are not aspirin*,* if there is doubt*,* I will be unsure of taking them*,* drugs have side effects.* (PwP2)

Where the PNS were accessible to PwPs over time, and a relationship could be developed, continuity of care was achieved, improving concordance.

*As we can talk about dose and time*,* adherence can be higher*,* consultants don’t give as much information*,* we talk through the rationale and why*,* I give a date they can ring me back to make sure there are not side effects*,* so it’s always ongoing follow up to ensure compliance.* (PNS1.4)

The inclusion of CPs in the reciprocal relationship with the PNS was also increasingly important for pharmacological management as the stages of Parkinson’s progressed and CPs took on more responsibility for treatment regimens.

*I have had one patient whose daughter stopped his tablets as they made him feel sick*,* so they reduced them themselves*,* but we could have given anti-sickness.* (PNS1.5)

For CPs and PwPs, it is the time available to PNSs which improves opportunity for concordance.

*In terms of [improved] compliance and adherence that could well be because sometimes nurses have a bit more time and ability in terms of explaining the efficacy and the reasons why we are giving the medications and how they work*,* and we have a bit longer to do that then the consultants.* (PNS1.1)

#### Concordance GT

Where PNS have the time available to provide continuity of care and to build trusting reciprocal relationships with PwP (and CP where appropriate) they can individually tailor pharmacological management and review to personal circumstances. Such trust and personalisation lead to improved concordance and so improved health and well-being.

**Sub-theme: speed of treatment**

Where PNSs were qualified independent prescribers, this sped up access to appropriate treatment for PwPs, improving symptoms and perceived well-being.

*When you need to titrate those drugs*,* you need to do it really quickly. If somebody is having side effects*,* you need to be able to do something about it today not in a weeks’ time. (PNS1)*

PNSs described various systems and practices they have developed to support PwPs as in-patients.

*We have electronic prescriptions and Get It On Time […] Parkinson’s UK showed that medications in hospital are being given often at the wrong time […] we now audit and get a monthly report of how timely wards are. [Our] whiteboard puts a symbol next to their name and it will stay grey when is not due and then turns green and then will flash and then will turn red if it is late. NICE guidelines say 30 min for doses*,* so the system follows that.* (PNS1.2)

Where PwP were involved with their PNS in treatment decisions, and they also had information from Parkinson’s UK, they had the tools to advocate for themselves about the importance of the timings of their medications as in-patients.

*I know it is important to be regular as there was a thing this year about meds on time as when I was in hospital in the morning*,* I was given a big pile of tablets*,* and they said*,* ‘here you go’ and I said ‘no*,* it has to be at times’.* (PwP12)

By prescribing, PNS’s could offer a more direct or even immediate route to medication.

*If you are not [a prescriber] there is a lot of admin in the background*,* so I know they need to change treatment but I need to write to a consultant and wait for them to get back and then have to write to GP*,* and they have to wait and the process has taken so long the patient has deteriorated and that has happened to me and the patient has gone off their legs. And it’s even worse if the consultant is on leave.* (PNS1.3)

PwP sometimes described complicated processes of pharmacological management which required significant resources of self-advocacy.

*This last [prescription] came from me ringing up and saying I am in a bad way and I need to see the doctor and [nurse] wrote a letter to my doctor as the hospital is not allowed to prescribe and apparently [GP] could argue*,* but [nurse] does that and the GP sends it to the chemist and that can take two weeks. If the change comes from [hospital name] it can take a month and I get apologies on my copied letter.* (PwP13)

By reducing the time waiting for appropriate medication changes, PNS can improve patient outcomes.

*As a prescriber you can do it there and then […] if they are waiting months [there is a] risk from falling [or] risk of swallow problems. So that window of risk shrinks if you can give them a prescription there and then and hopefully you are making them safer and their quality of life better as well.* (PNS2)

#### Speed of treatment (pharmacological support) GT

Where time is available to PNSs to advocate for PwPs, PNSs expertise in Pharmacological Management and Review allows them to develop and apply procedures and policies which ensure PwP receive the right medication at the right time when in acute care.

#### Speed of treatment (Pharmacological Access) GT

When PNSs are non-medical prescribers (NMP) they can review medication regimens and prescribe and stop medications as appropriate. This action speeds up treatment changes to PwPs, cutting through circuitous routes to medication management through other HSCPs.

### Overall grounded theory: the impact and value of the PNS

Drawing together all our findings and theories we suggest one overarching final theory of how PNSs can and do support PwPs and CPs.

See Fig. 1 for a visual overview of overall findings.

#### Final overall GT

Where a PNS has time, training, and support available to them, they can provide person-centred care to PwPs from diagnosis; including, expert counsel, a conduit of care, and pharmacological management and support through NMP status. Relationships of trust and partnerships can be built with both the PwP and their CP, improving responsivity and concordance, confidence and empowerment; ultimately, improving health and well-being.

### Key recommendations for the Profession and/or patient care

Building on our findings, we make nine key recommendations. For a more extensive list please see supplementary file 6.

1. PwP should be referred into PNS service at diagnosis and be supported to have regular appointments throughout the stages of their condition so PNS can offer on-going personalised specialist pharmacological support and management.
2. PNS need a manageable case load and administrative support. High caseloads and low levels of administrative support impede PNS to provide the time needed for person-centred continuity of care for PwP and CP to ensure positive outcomes.
3. PNS need specialist training in the management of people with Parkinson’s and their CP, to support them in their role, with a focus on person-centred care.
4. PNS must be available and accessible to PwP and their CP where required to provide specialist expert emotional, education, and lifestyle advice about this complex condition.
5. PNS provide significant referral and signposting to PwP which has a positive impact on their health and well-being. They require the opportunity to build these networks and the time to provide this conduit of care to PwP and CP.
6. PNS should work towards Non-Medical Prescriber (NMP) status early in their PNS career to build the confidence and extensive knowledge base required for this useful but complicated role. Pharmacological management and review should be incorporated into specialist nurse training and CPD.
7. PNS must be afforded the time to build relationships and partnerships with PwP and CP through continuity of care to encourage the sharing of pertinent information.
8. PNS use their specialist knowledge to provide informal and formal education to other Health and Social Care professionals (HSCP) which improves patient care. Time and resources need to be made available to PNS to continue this.
9. Appropriate job planning, and succession planning is required to protect the availability of PNS to PwP and CP.

## Discussion

Across the data, spanning insights from PNSs, PwPs, and CPs, was the appreciation of and importance placed upon personalised compassionate care from a respected specialist nurse. Where this could be established early, from diagnosis, this had the potential to facilitate sustainable positive outcomes for PwP and CP; that is, they could suitably manage their symptoms and sustain improved quality of life.

A recent international review of the PNS found they perform several models of personalised care, including proactive and process monitoring, care coordination, patient navigation, and provision of wide variety of information [8]. Yet, while it was clear that PD negatively impacts the lives of PwP and their CP, there was little evidence of the positive impact of the PNS [8]. Resultingly, we hope this addition to the PD literature, with direct evidence of care models and theoretical concepts, provides new verification on the value of personalised care from PNS, that is also expanded to include pharmacological management.

As the study described herein progressed, we identified that many elements of Santana’s [17] conceptual framework of person-centred care (PCC) were being described by participants, particularly where both PwPs, CPs, or PNSs were indicating positive outcomes. Their framework draws out different domains where action and behaviour can contribute to the delivery of PCC; structurally, workforce and cultural development is required to facilitate and embed PCC. While at the delivery level, cultivating communication, respectful and compassionate care, the engagement of patients in the management of their care, and integration of care, are all indicated as crucial for positive outcomes [17].

As others, our findings demonstrate the value of providing PCC. PCC comprises an authentic presence between professional and patient, while requiring both professional and personal empathy, the potential for which is improved with continuity of care [28]. PCC is underpinned by mutual respect, dignity, and empowerment [29]. We similarly found the importance of approachability of caring professionals to building trusting relationships that allowed for inclusion and reassurance [30]. Also, that positive examples of care correlate with the ‘six Cs’: care, compassion, competence, communication, courage, and commitment [31].

For the PNS to provide PCC, to both the PwP and the CP, requires time. This can be challenging since appointments are often time limited and, once the PwP has had the opportunity to discuss their needs, there is often little time left to explore the CP needs. The PNS is required to support two people in clinic ‘slots’ designed for one person. It is important that CPs are recognised as a vital and important member of the multidisciplinary team. Where they were included appropriately, the PNS could be alerted to potential problems, and/or the PNS and CP had space to pool information pertinent to the care of the PwP to improve their well-being. Such opportunity for exchange led to improvements in care for the PwP and reduced caregiver strain. While CP inclusion was clearly beneficial from the data, models of care in practice tend to only recognise the patient, despite much care and communication being directed to and received from the CP.

Compassion requires time and space; nurses require time with patients to encourage compassion [32]. Previous research has identified that nurses had compassion satisfaction when there was reciprocity, where they met their own expectations for care and their expectations of other HSCPs and organisations were met. Alternatively, they had compassion fatigue and became disillusioned if they could not get the resources to undertake their role to their expectations [33]. We name ‘compassion’ in the Continuity and Partnership GT due to the sharing of feelings and experiences such as “comfort”, and “understanding”, and the recognition of care given and taken. Compassion is unapologetically emotional and can also be subtle, existing in small kindnesses in what could be seen as simply routine and mundane tasks [34]. Relationships are built through these varied compassionate moments, earlier research challenges some of PCC, prioritising this reciprocity and placing the relationship (rather than the person) in the centre of care [35, 36]. Herein, we agree of the imperative of the relationships shared by all three participant groups.

PNS, PwP, and CP, all described how diagnosis could often be a shocking and difficult time. PwP often struggled to understand and accept their diagnosis. The provision of specialist information, education, and support at this early stage provided opportunities to reduce anxiety and distress, allowing PwP to ‘own’ their condition without overly relying on less appropriate or efficient section of the healthcare system (e.g. the GP). When the PNS becomes familiar with the whole family early in diagnosis and builds rapport, this allows them to accompany the PwP and CP throughout the Parkinson’s journey/trajectory. This companionship gives them an opportunity to mobilise education, support, listening and time, which can lead to families feeling that they are taking control of the situation and increases their confidence to manage and accept the condition. This provides a foundation for sustained self-care throughout their ever-changing journey.

Providing the intermediary role often relied on relationship building with other services; PNS instigated joint activity and information sharing, which was frequently alluded to by the PNS as good practice to ‘get things done’ for the PwP. We called this creating a ‘conduit of care’. Earlier research has shown that nurses identified themselves as ‘being a conduit’, wherein they facilitated and smoothed transitions, listening to concerns and acknowledging needs and joining-up services [37]. We found the value of the conduit role to be significant, involving connectivity and engagement. This required PNS to have a wide network to refer and signpost on to, but also to have a knowledge of the individual and their condition specifically. This is a skilled endeavour, relying on significant knowledge together with time.

PwP explained the value of referrals, signposting, and contact with other HSCPs, departments and agencies; helping them to negotiate difficult systems and services in difficult circumstances. Where this role of conduit was unavailable to a PwP (when they did not a PNS), the lack of co-ordination had a negative impact. PwP in this position described long processes of referrals to multiple parties before problems were addressed, if at all. Overall, our findings found there was extensive evidence of the comprehensive nature of the conduit of care approach taken by PNSs to PwP, and that where this was appropriate, this was also extended to CP with positive outcomes for them and the PwP, including improved engagement with services, improved knowledge, and improved well-being.

Similar extensions of these boundaries of care were outlined where PNS described supporting PwP to negotiate their way through other services, advocating on their behalf for them and providing tools to self-advocate. PNS also described (in)formal education practices, incorporated into their workload. These forms of training require time for building extensive network contacts, including with HSCPs who may not be immediately receptive to the message, as well as the skills to produce and deliver this education and training. The purpose of this training was described as a method of ensuring that PwP were treated appropriately as they encounter these different services and as such, this form of education was a pro-active attempt to improve outcomes and experiences for PwP negotiating different services.

When the PNS is a qualified independent NMP, they can promptly respond to PwP pharmacological needs and actively involve PwP in review and support, so ensuring informed actionable decisions are made about their treatment. This approach to care makes PwP feel understood and included, leading to improved concordance, and so improved symptom control and PwP well-being. Concordance, rather than compliance or adherence, is used herein as the former terms signify a more paternalistic approach to care. Rather, concordance recognises partnership in care [38] and it is the value of the relationship built with the PNS which generates the positive outcomes from the specialist nurse undertaking NMP. Experienced nurses demonstrate advanced communication and assessment skills [39] and the opportunity to provide more holistic and comprehensive treatment plans [40], all of which provide a secure basis for NMP. Our findings further demonstrate that these relational competencies build trust and inclusion, encourage partnership working, and provide the space for medication regimens to be appropriately discussed and tailored to individual circumstances, ultimately improving concordance.

Where PNS were NMP they were able to improve speed of treatment to PwP. Where nurses were not able to prescribe, circuitous and time-consuming routes were often required to get medication changes made. These often-necessitated significant amounts of (self) advocacy, created gaps in response, and the opportunity for lost and miscommunication between professionals and to PwP and CP. As others, we found nurse prescribers could improve continuity of care, improving patient satisfaction and their own job satisfaction, and reducing wait times and potential for hospital admissions [40, 41].

The ability to independently prescribe is only granted following the successful completion of an accredited university non-medical prescribing programme, registering this qualification with the Nursing Midwifery Council (professional body of the PNS) and then authorisation from the employer (with inclusion of prescribing within an individual’s job description) to use this extended skill. If the PNS has not had sufficient training, support, or supervision to treat or manage specific conditions or symptoms then their sphere of prescribing competence will be limited. Improving the opportunity for nurse prescribing requires appropriate prescribing policies and good access to continuous professional development. As others, we found facilitators include good education, supervision, good working relationships with medical staff and pharmacists [42].

Nurses can be put under pressure to prescribe, from patients and other HSCPs, and boundaries around prescribing can become blurred [43, 44]. Our research also identifies tension on occasion with other HSCPs around boundaries of NMP. Workplace policies and procedures should clearly outline the value and purpose of nurse prescribing to ensure their competencies are recognised and remain accessible. Further to this recognition, prescription is time-consuming, nurses carefully audit and document these tasks [45] and time needs to be provided appropriately in caseloading for the administration of this aspect of the specialist nurse role [41]. Joined up systems are required to ensure that nurses have full access to information pertinent to patients before undertaking pharmacological management and review [42].

Specialist nurses are highly skilled and competent staff, encouraging nurse prescribers therefore allows for the full use of their competencies [40]. While there is an economic imperative for all the health and social care workforce to be working to their full capacity and scope of practice [39]. NMC standards assert NMP should only prescribe within their own scope of practice and recognise the limits of their own knowledge and skill [46]. This limited range is different to the medical model of prescription, and so can produce tensions for patients with co-morbidities. Trust can be built to overcome some of this tension through the recognition of speciality [47]. PNS in our study, by dint of their specialist knowledge of the complexity and individuality of PD, could inspire confidence and reassurance and so establish trusting working relationships with medical professionals.

This study was undertaken in the UK, where nurses have been prescribing for an extended period, yet the practice of NMP is increasing worldwide, particularly across high income countries [39]. Our findings contribute to the evidence base as this practice increases worldwide, specifically demonstrating the value to patients and wider medical teams where nurses can prescribe within their speciality.

The PNS, through relationships, partnerships, and expertise, can reduce the ‘burden of treatment’ on the PwP and CP through the provision of responsive, pro-active, and personalised care and support [48]. Through what we characterise as ‘working together’ and a ‘conduit of care’, PNS can meet PwP where they are, recognising individual contexts, social capital, and skills, to bridge the gap to services for those with a complex long-term condition and improve inclusion. The PNS was the preferred person/professional for contact and engagement across all data categories. Where appropriate resources were available, PwP and their CP felt they were ‘working together’ as part of a team or partnership with their PNS. This built a relationship of trust over time and allowed for reciprocity and reassurance. As others, we identify the establishment of partnerships of shared responsibility [16]. Our findings corroborate the benefit of an integrated person-and-community centred approach involving several sectors [49]; identifying the value of providing PCC, the provision of a ‘conduit of care’, and ‘working together’ in Parkinson’s nursing.

### Limitations

Findings should be considered against the following limitations. Due to COVID-19 impact on project time and production, findings have taken longer to write-up than we would have preferred. There are significant challenges in evaluating the PNS role as they often work within MPTs, with different service models and therefore attributing outcomes to the PNS alone is challenging. PNS cover most of the UK, making it neither possible nor ethical to establish a matched control group of PwP that do not have access to a PNS, against which to compare outcomes. While we did have a relatively large sample size, due to the complexity of different services and delivery models, it was not possible to model different approaches in as much detail as we envisaged at project conception. We were pleased to get such a significant response to invitation to participation, for which we thank our networks and PPI representatives. This gave us a strong base from which to undertake selective sampling of interviews to avoid positive bias. This sampling ensured a range across those who indicated they did /not have a good relationship with their PNS, as well as those in various stages of Parkinson’s, with different demographics, and differing socio-economic circumstances. Subsequent research to further test and investigate in as many communities as possible would be beneficial.

As others point out, even with several coders it can be difficult to ensure that researchers do not fall too easily into agreement and consistency rather than robust disagreement to discover aberrations [50]. We hope we have minimised this through drawing together a research team with a range of experience of research and Parkinson’s, who met regularly to discuss emergent findings in an open and supportive environment. These meetings included open and challenging discussions which built in rigour [51]. Similarly, the range of knowledge and experience with Parkinson’s and Parkinson’s services between the researchers was so significant it lent into reflexivity. The researcher with the least prior knowledge, from a social science research background with no nursing experience, (KM) led the data collection and analysis. While our researcher with the most clinical expertise, an experienced Nurse Consultant in PD (AH) deliberately followed rather than led through this part of the research. While this likely added time onto the study we embraced this as we found it brought credibility to the emergent theories and categories [52].

Through the research design, which adopted an interpretivist constructivist approach, we aimed to examine in-depth the ‘contextualised human experience’ [53], and so sought theoretical generalisability and transferability. Further, our emergent grounded theories and subsequent final grounded theory are all increasing abstractions of the general from the particular, which as they are also aligned to substantive theory in PCC, allow us to make claim to generalisability of our findings [53].

## Conclusion

Our research strongly suggests that every PwP should have access to a PNS from diagnosis. Further, that they should retain relationships with PNS and PNS services across the stages of PD to provide continuity of care. Skilled PNSs can provide expert counsel, which includes emotional support, education, and lifestyle advice, which can be shared with PwPs and CPs where appropriate. PNSs that provide compassionate person-centred care provide reassurance, building trust and confidence in PwPs and their CPs to manage their condition. The long-term relationships allow for partnership working, wherein PwPs, CPs, and PNSs ‘work together’ in a reciprocal way. PNSs provide a ‘conduit of care’ to PwPs, CPs, and other HSCPs, improving knowledge exchange, access to care through signposting and referrals, engagement with other appropriate services, and improving other HSCPs awareness of PD.

Parkinson’s nurses have the specialist skills and competencies to prescribe complex medications to PwPs. PNSs have the time (in opportunity to offer continuity of care and in longer appointments) and built trusting relationships with PwPs and their CPs to ensure personalised care and so appropriate actionable treatment regimens. Where PNSs can prescribe, PwPs have improved concordance due to better understanding of the medications issued that best suit their individual circumstances. Where PNSs can prescribe, speed of treatment to PwPs is significantly improved, reducing negative outcomes for PwPs, and avoiding circuitous routes to treatment through other HSCPs. PNSs should be supported to undertake NMP qualification from an early career stage, and continually supported through education, training, and workplace policies and procedures to work to the full scope of their practice.

PNS identified barriers to care such as time pressure due to education and training, a high caseload, and a lack of administration support. Facilitators include the competency to build and develop their role, the need for appropriate job planning in line with competencies, and administration support.

## Electronic supplementary material

Below is the link to the electronic supplementary material.

Supplementary Material 1

Supplementary Material 2

Supplementary Material 3

Supplementary Material 4

Supplementary Material 5

Supplementary Material 6

## Data Availability

Availability of Data and Materials The datasets used and/or analysed during the current study are available from the corresponding author on reasonable request.The interview data herein have not been published elsewhere.
