# Supplementary material for: The impact and value of the Parkinson’s nurse specialist to people with Parkinson’s and their care partners: a grounded theory qualitative study
Source: BMC Nurs. 2024 Oct 28;23:791. doi: 10.1186/s12912-024-02441-7 (PMC11520507; doi:10.1186/s12912-024-02441-7)
Supplement: Supplementary file 1 — Supplementary Material 1 [file 12912_2024_2441_MOESM1_ESM.docx]

| **Interview Topics (condensed) PwP/CP** | **Interview Topics (condensed) Nurses** | **Codes** | **Data Categories/(Sub) Categories** | **Grounded Theory** | **Recommendation** |
| --- | --- | --- | --- | --- | --- |
| Where (else) do you get emotional support, education, lifestyle advice from?  What difference does it make? | Value of emotional support? Examples? Impact on role?  Education impact self-management? How, why difference?  Time spent (in)formally educating?  Lifestyle advice? Examples?  Examples of supporting CP and outcomes? | Emotional support – time/knowledge/relationship/trust; communication skills; consistency/continuity; connection; reassurance; missing; another HSCP.  Education- social media/websites complexity and incorrect info; what questions to ask; relationship/trust to ask/hear; uncertainty/fear; phone/f2f/groups; re/pro-active; stage appropriate/timing; individually appropriate.  Lifestyle advice- actionable; personalised; responsibility; builds on education; requires relationship/trust.  CP Inclusion- increases information (real/honest/pertinent); actionable; support; time; resources; team; include/ exclude; preference; carer strain. | **Data Category 1**  Expert Counsel; Emotional, Education, Lifestyle  From Diagnosis  All Stages | **Emotional Support GT:** When PNS have available time (within an appointment and in caseload to meet regularly) they can utilise their communication skills to provide individualised and authentic emotional support to PwP and CP which improves perceived well-being.  **Education and Lifestyle GT:** When PNS have available time (within an appointment and in caseload to meet regularly) they can share personalised education and lifestyle advice that provides PwP confidence and reassurance, this empowers them to manage their Parkinson’s, improving their health and well-being.  **CP Inclusion GT:** CP provide on-going and increasing modes of care to PwP. Where they are included in PNS expert counsel, they can help ensure personalised care, better supporting the PwP, and receive advice, support and guidance which reduces carer strain.  **Diagnosis GT:** Diagnosis can be a stressful time, PwP require access to a PNS and their expert counsel from diagnosis to improve their confidence and ability to manage their Parkinson’s. | 1. PNS must be available and accessible to PwP and their CP where required to provide specialist expert emotional, education, and lifestyle advice about this complex condition.  2. Every PwP should have access to a PNS from diagnosis.  3. PNS require the opportunity to build knowledge and communication skills in line with competencies to deliver expert support to PwP and CP. |
| **Interview Topics (condensed) PwP/CP** | **Interview Topics (condensed) Nurses** | **Codes** | **Data Categories/(Sub) Categories** | **Grounded Theory** | **Recommendation** |
| What services do you access? How, when?  What does (not) work in accessing?  Who, What is your key ‘go to’- what difference, impact does this have? | Signposting, referrals, links? What (does not) work?  Benefits, challenges?  Key ‘go to’ (how) does this or could this work? | Referral/Signposting- appropriate; no/slow; patient-led; external access; engagement; requests; availability; GP supports/barrier; relationships.  Accessibility- clear routes; phone/video/f2f; GPs avoid/preference/poor access; time; diagnosis (late/difficult/positive); contact difficult.  Key ‘go to’- hub and spoke; accessibility; consultant positive/negative; GP positive/negative; specialist; relationship/trust; resources | **Data Category 2**  Conduit of Care- PwP  Conduit of Care- CP  Barriers, Facilitators to PNS, Care, Support | **Conduit of Care to PwP and CP GT:** PNS provide a ‘conduit of care’ to PwP and CP, their extensive knowledge of Parkinson’s Disease, an individual Parkinson’s, and their networks and relationships with other HSCPs and agencies, allow them to facilitate appropriate referrals and signposting and the encouragement to engage.  **Conduit of Care to HSCP GT:** PNS provide a ‘conduit of care’ to HSCP through extensive (in)formal education to ensure better care for PwP as they encounter them. | 4. PNS provide significant referral and signposting to PwP which has a positive impact on their health and well-being. They require the opportunity to build these networks and the time to provide this conduit of care to PwP and CP.  5. PNS use their specialist knowledge to provide informal and formal education to other HSCP which improves patient care. Time and resources need to be made available to PNS to continue this. |
| **Interview Topics (condensed) PwP/CP** | **Interview Topics (condensed) Nurses** | **Codes** | **Data Categories/(Sub) Categories** | **Grounded Theory** | **Recommendation** |
| What has Parkinson’s journey been like so far?  When have you needed support?  Did you receive it? What did this mean? | Transition. Stages support – experiences, examples.  MDT working? | PwP/Nurse Partnership- inclusive; relationship/trust; partnership; time constraints; space for query/discussion; engagement; remote; other HSCP; loses impetus/engagement; interactive; transactional; consistency/continuity; active participant; empowerment; wider team; advocacy.  MDT- in/formal; access; referral/signposting; specialism; connection; engagement; support.  Monitor/Manage- too much?; too little?; consultant; GP; organising/advocacy; troubleshoot; accessibility; trust; self-medicate/stop/change by PwP or CP for PwP; reassurance; confidence.  Transitional/Stages- positive; negative; planning; reactive; proactive; relationship/trust; consistency/continuity; joint decision-making; engagement; inclusion/prepared-ness; reassurance. | **Data Category 3**  Team- Partnership- ‘Working Together’ | **Continuity and Partnership GT:** Parkinson’s is a complex life-long condition where a PwP will transition over several stages, each bringing different challenges. Where a PwP and their CP can build a relationship with a PNS, they can better manage changes and feel reassured they have a compassionate companion.  **'Working Together’ GT:** Where PNS have the time and competencies to provide person-centred care, a partnership is developed between PwP, CP, and PNS. This partnership, requiring continuity of care, allows for the exchange of pertinent information which can improve health and wellbeing outcomes. | 6. High caseloads and low levels of administrative support impede PNS to provide the person-centred continuity of care for PwP and CP to ensure positive outcomes.  7. PNS must be made available to PwP and CP to provide person-centred continuity of care across the stages of Parkinson’s.  8. PNS must be afforded the time to build relationships and partnerships with PwP and CP through continuity of care to encourage the sharing of pertinent information.  9. Appropriate job planning, and succession planning is required to protect the availability of PNS to PwP and CP. |
| **Interview Topics (condensed) PwP/CP** | **Interview Topics (condensed) Nurses** | **Codes** | **Data Categories/(Sub) Categories** | **Grounded Theory** | **Recommendation** |
| Who do you talk to about medications? Who makes changes? What (does not) work? What is best way for you to get medications? Who monitors? When/How? What are consequences? | Benefits/challenges of prescribing and titration?  Reflect on impact on concordance/speed of treatment/nurse knowledge-medication and PwP? | Adherence; medication + patient; speed of treatment; community/hospital pharmacist; GPs barrier/facilitator; improve connections liaising other HSCPs; (missed) reviews; waiting for consultant; responsibility; access; information; continuity; explanation and rationale; concordance; uncertainty; self-medication; dosage/timings; specialism- Parkinson’s; specialism/expertise in individual’s Parkinson’s; engagement. | **Data Category:** Pharmacological Management and Review  **Sub-Categories:** 1. PNS (Specialist) Prescribing; 2. Concordance; 3. Speed of Treatment | **Pharmacological Support GT:** While not all PNS prescribe, most recognise the value of non-medical prescribing (NMP) to professionals, patients, and wider team functioning, and are keen to work towards this status. Yet NMP is a complex and responsible position which requires confidence building, specialist training, and understanding of the parameters of the role in wider teams.  **PNS (Specialist) Prescribing GT:** PNS are specialists in Parkinson’s Disease, Parkinson’s medications, and an individual’s Parkinson’s (which can be extremely variable). Consequently, they can provide personalised pharmacological support with positive impacts on PwP health and well-being.  **Concordance GT:** Where PNS have the time available to provide continuity of care and to build trusting reciprocal relationships with PwP (and FFC where appropriate) they can individually tailor pharmalogical management and review to personal circumstances. Such trust and personalisation lead to improved concordance and so improved health and well-being.  **Speed of Treatment (Pharmacological Support) GT:** Where time is available to PNS to advocate for PwP, PNS expertise in Pharmacological Management and Review allows them to develop and apply procedures and policies which ensure PwP receive the right medication at the right time when in acute care.  **Speed of Treatment (Pharmacological Access) GT**: When PNS are non-medical prescribers (NMP) they can review medication regimens and prescribe and stop medications as appropriate. This action speeds up treatment changes to PwP, cutting through circuitous routes to medication management through other HSCPs. | 10. PNS should work towards NMP status early in their careers to build the confidence and extensive knowledge base required for this useful but complicated role. Pharmacological management and review should be incorporated into specialist nurse training and CPD.  11. PwP should be referred into PNS service at diagnosis and be supported to have regular appointments throughout the stages of their condition so PNS can offer on-going personalised specialist pharmacological support and management.  12. To improve PwP concordance with medication regimens, PNS require time (in caseloads and regional availability) to build reciprocal trusting relationships with PwP.  13. PNS must be accessible and available to support medication management to PwP in acute care to ensure they get their treatment regimens are not disrupted.  14. PNS should be qualified NMP to ensure speed of treatment to PwP to improve health and well-being outcomes.  15. PNS require appropriate time to be included in caseload to manage medications given this is a task comes with a high administration burden to ensure patient safety.  16. PNS are recognised specialists but boundaries between medical and non-medical prescribers can become blurred and tense if not managed. Workplace policies and procedures should ensure the PNS role is clear and well-supported. |
