# Supplementary material for: The impact and value of the Parkinson’s nurse specialist to people with Parkinson’s and their care partners: a grounded theory qualitative study
Source: BMC Nurs. 2024 Oct 28;23:791. doi: 10.1186/s12912-024-02441-7 (PMC11520507; doi:10.1186/s12912-024-02441-7)
Supplement: Supplementary file 2 — Supplementary Material 2 [file 12912_2024_2441_MOESM2_ESM.docx]

| Data Category + Sub-Category | Code | Code | Code |
| --- | --- | --- | --- |
| (Data Category 1) Expert Counsel – (Sub-Category 1) Emotional Support (is desired/provided by a PN that is available and accessible) | time/knowledge/relationship/trust | Reassurance + Connection | Reassurance + Connection |
| *It would be amazing to have someone who understands who you could go to [with] the things you don’t want to talk to the consultant about [and] the GP doesn’t understand, would be massive both emotionally and physically [...] we would just like to ask what do you do with this? [...] but we are just left on our own and you feel like you are floundering. (CP3)* | *[Emotional support] is really really important. I suppose sometimes it is more important than managing symptoms for some people. Patients develop a relationship with you and they develop a trust and they know if they have problems they can approach you in confidence. They often come with lots of problems which are not related to Parkinson’s and often talk about family issues and you can get wrapped up in a lot of that but is important as their condition affects it. (SN3)* | *[when had nurse she] would talk to mum about how to make things better and boosting her emotionally and her efforts and what she as still achieving and doing. (CP2)* | *if you were feeling a bit down or sorry for yourself [nurse] would bolster my confidence and [gave advice on what to do and how], I could phone her, [and] even if I didn’t [phone], it was knowing there was somebody there you trusted you could contact, and she knew me as a person as I had known her since initial diagnosis and your world falls apart when you get your diagnosis and she helped me put it in perspective and [contact with nurse] helped me put it into perspective and that I was the same person as before and things could just continue. (PwP2)* |
