# Supplementary material for: The impact and value of the Parkinson’s nurse specialist to people with Parkinson’s and their care partners: a grounded theory qualitative study
Source: BMC Nurs. 2024 Oct 28;23:791. doi: 10.1186/s12912-024-02441-7 (PMC11520507; doi:10.1186/s12912-024-02441-7)
Supplement: Supplementary file 3 — Supplementary Material 3 [file 12912_2024_2441_MOESM3_ESM.docx]

Adapted from Berthelsen, Grimshaw-Aagaard, & Hansen (2018, Table 3, p.70) The Guideline for Reporting and Evaluating Grounded Theory research studies (GUREGT) Using Charmaz.

| **Area** | **Item** | **Grounded Theory Methodology- Charmaz** | **Identification** |
| --- | --- | --- | --- |
| Study Aim | 1 | Is the grounded theory study aim presented to construct a theory focusing on examining process and actions? | In section 1 ‘background’ and section 2 ‘methods’ we identify our intention to generate explanatory theories following investigation of social process and practices within the field of Parkinson’s Specialist Nursing. |
| Philosophical framework | 2 | Is the grounded theory embedded in symbolic interactionism and social constructivism? | In section 2 ‘methods’ we identify we are specifically seeking in the data to recognise and understand patterns of behaviour and actions within contexts in line with social constructivism. This GUREGT guideline is specifically chosen for Charmaz (2006, 2014, 2016) approach. |
| The researchers’ role | 3 | Is the researchers’ reflective and interpretive stance in a two-way interaction with the participant described and explained? | In section 2 we identify how the researchers worked together to consider and challenge each other’s reflexivity throughout data collection and analysis ensuring this was maintained.  In section 2 we also identify how the topic guides were reflexive and adapted through theoretical sampling. |
| Data collection | 4 | Are data collection methods described and explained? | See section 2 we identify semi-structured interviews were used that followed emergent theorising and allowed for participant voice. Researchers met regularly to discuss interviews as they developed. |
|  | 5 | Has qualitative or quantitative data collection methods been used? How and why? | Qualitative data collection methods were used to allow for participant voice and in-depth interrogation of a wide range of experiences, actions, and ‘value’ of services (not) offered. |
| Memos | 6 | Have memos been written throughout the study about concepts and categories and are they used to formulate and construct the theory? | Using MS Word, in online meetings and through conversations and reflections of the data regularly throughout the data collection and analysis memos were written to consider (potential) codes, categories, (conflicting) incidences to generate and refine theories. |
| Sampling procedures | 7 | Is initial sampling, conducted in the beginning of data collection, described and explained? | We recruited online through wide networks and promotions with stakeholders. This is described in section 2. |
|  | 8 | Is theoretical sampling of the emerging categories and theory from the data collection described and explained? | We have explained our selective and theoretical sampling approach in section 2. We have provided a supplementary file (1) which demonstrates the development of the sampling, data collection, and theory. |
|  | 9 | Is the selection of participants guided by theoretical sampling? How? | As above, we ensured we reached a wide range of participants were engaged and guided the Selective and then subsequent theoretical sampling to ensure multiple circumstances were included and we were able to follow the data. |
| Theoretical saturation | 10 | Is the reach of theoretical saturation explained according to no new insights relevant for the concepts and categories and the emergent theory? | Yes, see section 2 for details of iterative analysis and emergence practises. Also, our explanation of the process of theoretical saturation used. |
| Analysis and coding | 11 | Is the coding levels and concurrent process of coding described according to initial, focused and theoretical coding? | See section 4.4 to explain the iterative and extensive analysis which was undertaken throughout both study phases to develop initial coding, to guide data collection tools, into focused coding to clarify and compare, finally into final theoretical coding. Also, how we sought meaning saturation. |
|  | 12 | Which codes have guided the specific coding levels and how? | Our codes included behaviours, actions, contexts of incidences – all can be seen in Supplementary File 1 and 2. |
|  | 13 | Is the basic social process identified before conducting focused coding? | As above. |
|  | 14 | Which theoretical codes have structured the theory to a progressive level of abstraction? | As above. |
|  | 15 | Is the constant comparison method used to compare incidents with incidents, incidents with categories and categories with categories? | As above and see section 2. |
|  | 16 | Is the simultaneous data collection, analysis and coding guided by the theoretical sampling and writing memos described and explained? | As above and sections 2. |
| Review of literature | 17 | Is the literature reviewed initially in the grounded theory study to expand the contextual framework? Why and how? | A comprehensive intro and background around Parkinson’s Specialist Nursing is provided in sections 1. As Charmaz (2006, 1014, 2016) grounded theory approach recommends- theoretical literature review was conducted as theories emerged (rather than before) to avoid influencing the analysis. |
|  | 18 | Is the literature reviewed during theory development on the basis of the emerging concepts and theory? How and on what grounds? | From the wider literature, person-centred care (PCC) became clear as an integral theoretical approach and explainer at a higher abstract level as the project progressed – this is outlined in discussion in section 4 ‘discussion’. |
| Results/ the theory | 19 | Are the main social interactions of the theory presented and explained? | Each data category sub-category has a clearly identified final GT that has emerged through the iterations of analysis and identification of the social contextual, processes, actions of all participants from the qualitative data. See section 3 ‘results’. |
|  | 20 | Is the basic social process and the related categories presented and explained? | As above |
|  | 21 | Does the theory account for the essential processes and actions in the social interactions of the participants? | As above |
|  | 22 | Are quotes used and argued for to describe the theory? | Yes, see section 3 for extensive qualitative quotes that build the final theory in each category. |
| Discussion | 23 | Are the key relationships between the categories and codes discussed and related to relevant literature? | Yes, this is covered in section 4 ‘discussion’, |
| Evaluation criteria | 24 | Are the criteria of credibility, originality, resonance, and usefulness, as well as fit, work, relevance, and modifiability presented and explained? | Charmaz approach is specified through section 2. Data is drawn from an original empirical study and is situated in the field in Section 1 and 4. We make several recommendations for resonance, usefulness, and relevance.  Within word count constraints we have sought to provide methodological detail alongside findings and discussion detail to allow for credibility and modifiability. |
|  | 25 | Are the evaluation criteria used to evaluate the theory? | As above. |
