# Supplementary material for: The impact and value of the Parkinson’s nurse specialist to people with Parkinson’s and their care partners: a grounded theory qualitative study
Source: BMC Nurs. 2024 Oct 28;23:791. doi: 10.1186/s12912-024-02441-7 (PMC11520507; doi:10.1186/s12912-024-02441-7)
Supplement: Supplementary file 4 — Supplementary Material 4 [file 12912_2024_2441_MOESM4_ESM.docx]

| **Participant** | **Quote** | **Code** | **Sub-Category** |
| --- | --- | --- | --- |
| PNS | *a consultant asked me to go out to see a lady and [I found on home visit that] she put all her tablets into a bowl, and she just would select what she wanted and that would never have been picked up if we hadn’t been able to go out and they just hadn’t been able to work out what the problem was* (SN11) (PNS) | Medication + patient | PNS (Specialist) Prescribing |
| PNS | *the CCG changed boundaries and they didn’t employ a nurse they employed a prescribing physio and they were making medication recommendations that I would say where above and beyond his experience and ended up in a few patients coming into our local hospital and they came in with the falls from low blood pressure and thankfully they realised they had a problem and changed to a nurse* (SN14) (PNS) | specialism- Parkinson’s | PNS (Specialist) Prescribing |
| PNS | *sometimes when people come to clinic it is not always about increasing doses, timings, interactions, it is about getting the medication to work as well as it possibly can. it is looking at diet or the combination of drugs they are taking just for instance if someone is taking iron, putting iron and levodopa together is going to affect the absorption and they are not going to work well together* (PN1) (PNS) | specialism- in an individual’s Parkinson’s | PNS (Specialist) Prescribing |
| PwP (has PNS) | *A year ago they did an extra tablet a day and she said to take it at night and I found I couldn’t sleep so I adjusted the day ones as I was often busy in the morning so I get up at 7 o clock and I put another tablet in there and I told her and she said oh no problem they like to do it adjust it to suit me* (PwP8) (PwP)  *Dosages and timing is absolutely better due to Parkinson’s nurse involvement. So, she is thorough and effective […] means more likely to take things as needed.* (PwP14) (PwP) | dosage/ timings | Concordance |
| PwP (no PNS) | *I phoned Parkinsons UK helpline as […] I had a telephone appointment not with consultant but one of registrars who upped my medication and added things and I didn’t know how to take them so I googled it and then called Parkinsons UK and l spoke to their nurses, […] they are powerful drugs and they impact on your body, […] if there is doubt I will be unsure of taking them, drugs have side effects* (PwP2) (PwP) | self-medication | Concordance |
| PNS | *I do start treatment and titrate and the benefit is that the patients symptoms are managed more effectively in a quicker time frame and lots of monitoring might be needed but I have the extra slots if it needs fine tuning and peak time regularly for a while and then draw back so the advantage is some patient will not be seen for a year by a consultant and they are being seen sooner and managed earlier and that can reduce falls and admissions and it hopefully helps symptom management enormously.* (SN13) (PNS) | waiting for consultant | Speed of Treatment |
| PwP (no PNS) | *I had an alteration of medication from the consultant it took me two weeks with 6 phone calls to GP and 4 phone calls to secretary to get the prescription sent to the pharmacist [when I had a PNS] the Parkinson’s nurse, when I had to have a change of medication and by the time I got home she had spoken to surgery and consultant and the script went across the next day*. (PwP11) (PwP) | GPs barrier/facilitator | Speed of Treatment |
