# Supplementary material for: The impact and value of the Parkinson’s nurse specialist to people with Parkinson’s and their care partners: a grounded theory qualitative study
Source: BMC Nurs. 2024 Oct 28;23:791. doi: 10.1186/s12912-024-02441-7 (PMC11520507; doi:10.1186/s12912-024-02441-7)
Supplement: Supplementary file 5 — Supplementary Material 5 [file 12912_2024_2441_MOESM5_ESM.docx]

Following is a merged document of all the following interview schedules:

PwP Interview Schedule (No Nurse)

PwP Interview Schedule (Nurse)

CP Interview Schedule (No Nurse)

CP Interview Schedule (Nurse)

PNS Interview Schedule

**PwP Interview Schedule (No Nurse)**

**Commence recording as soon as you begin the interview** – including the preliminary discussion.

**Step1: Introduction**

“Good Morning/Afternoon/Evening. **Thank you for agreeing to be interviewed. My name is Katy McEwan. I** work at Northumbria University and am one of the researchers on the USP study.

The purpose of this study is to explore scope and value of the Parkinson’s Specialist Nurse.”

“**Please could I check that you have read and understand the information sheet?** Just to reassure you that everything you say is confidential and you will not be named in any report/paper to come out of the study. Only the research team and transcriber will have access to the recording, which will be destroyed once transcribed. It’s likely that the interview will take between 45 and 60 minutes but please let me know if you’d like to stop at any time for a break or if you would prefer to stop the interview completely. And to reiterate, you are free to withdraw from the study at any time without giving a reason.”

Please obtain verbal consent to participate- **“Can I confirm that you consent to take part in the interview and for the interview to be recorded?”**

“To help you understand what to expect – there are a couple of introductory questions then following questions come under three different themes– I will let you know when we move between sections, so you are aware of the pace and where we are in the interview.”

“**Have you any questions before we start**?”

**Step 2: Interview**

**General**

- (If has…) Can you tell me what your supporter would like to be called?
- We will talk about this in different ways in the upcoming questions, but generally, how do you feel about services for People with Parkinson’s?

**Section One**

Explain:

*We are wondering how different aspects of the counselling element in the relationship between the PwP and the nurse works and how it impacts on quality of life and interaction with other services. The first section of questions is specifically related to how you feel that might work in practice.*

**1.1**

- Where do you receive emotional support from?
- What difference does this make to your quality of life and day to day living?
- Do you think the emotional support a nurse could offer would be beneficial? How so?

**1.2**

- Where do you receive education about your Parkinson’s from?
- Could you share any examples of what you have learnt and the difference it has made to you?
- Would it make a difference if you could access education from a Parkinson’s nurse? How so?

**1.3**

- What is the difference between ‘education’ and ‘lifestyle advice’?
- Where do you get ‘lifestyle advice’ from and what difference has this made to you?
- Would you expect to get lifestyle advice from a Parkinson’s nurse?

**1.4**

- Who supports you through your Parkinson’s journey?
- Who supports them?

**Section 2- Access**

Explain:

*We are wondering how different elements of increased access to specialist services works and what impact this has. The following questions are specifically related to how you feel this works in practice for you and how you feel about access.*

**2.1**

- What services do you access inside or outside of the NHS to support you in your health and wellbeing?
- How did you find out about them?

**2.2**

- How do you contact the services and providers of your care?
- What does(n’t) work when you do this?
- Do you travel to appointments? How do you do this? Can you tell me about who comes with you?

**2.3**

- Do you have a key ‘go to’ person for your Parkinson’s care?
- What would you be likely to contact them for? And what would you choose to go somewhere else for?
- Does having a ‘go to’ person reduce the number of different contacts and appointments you need to make? Can you think of an example?

**Section 3- Medication and Monitoring**

Explain:

*We are wondering how medication and monitoring works across difference service models and what the impact of these are on PwP. The following questions are specifically related to how you feel this works in practice for you and how you feel about medication, prescription, and monitoring.*

**3.1**

- Who do you talk to about your Parkinson’s medication?
- Who discusses medication changes with you- why changes need to be made and what that will mean for you?
- Do you understand all the medications you are on and why?
- Do you always take your medication as prescribed? Could you explain why?
- Who issues your prescriptions?
- Where do you pick up your medication from and who helps you?
- Is this the best way for you? (what’s ideal and why?)

**3.2**

- Who currently monitors your Parkinson’s?
- How often do you see them?
- Do you have to ask for additional help or troubleshoot problems with anyone? Can you share any examples of what that means for you?
- Do you think it is possible that you could be offered ‘too much’ support from a anyone? Is there such a thing as ‘too-much’? Could you explain why you think this?

**3.3**

- Can you tell me about your Parkinson’s journey so far and when you have most needed support?
- Who has given you support and care at different times and what has this meant to you?

**Step 3: Closing**

**“We’ve completed all the planned questions.** **Is there anything you would like to add before we finish?**

Thank you very much for your contribution. Please feel free to contact me again if there is anything more, I can share about the study.

**Would you like to receive a summary of the outcomes of the study?”**

State time/date for the recording.

**PwP Interview Schedule (Nurse)**

**Commence recording as soon as you begin the interview** – including the preliminary discussion.

**Step1: Introduction**

“Good Morning/Afternoon/Evening. **Thank you for agreeing to be interviewed. My name is Katy McEwan. I** work at Northumbria University and am one of the researchers on the USP study.

The purpose of this study is to explore scope and value of the Parkinson’s Specialist Nurse.”

“**Please could I check that you have read and understand the information sheet?** Just to reassure you that everything you say is confidential and you will not be named in any report/paper to come out of the study. Only the research team and transcriber will have access to the recording, which will be destroyed once transcribed. It’s likely that the interview will take between 45 and 60 minutes but please let me know if you’d like to stop at any time for a break or if you would prefer to stop the interview completely. And to reiterate, you are free to withdraw from the study at any time without giving a reason.”

Please obtain verbal consent to participate- **“Can I confirm that you consent to take part in the interview and for the interview to be recorded?”**

“To help you understand what to expect – there are a couple of introductory questions then following questions come under three different themes– I will let you know when we move between sections, so you are aware of the pace and where we are in the interview.”

“**Have you any questions before we start**?”

**Step 2: Interview**

**General**

- (If has…) Can you tell me what your supporter would like to be called?
- We will talk about this in different ways in the upcoming questions, but generally, how do you feel about services for People with Parkinson’s?

**Section One**

Explain:

*We are wondering how different aspects of the counselling element in the relationship between the PwP and the nurse works and how it impacts on quality of life and interaction with other services. The first section of questions is specifically related to how you feel that might work in practice.*

**1.1**

- Does your nurse provide you with emotional support? Can share any examples?
- What difference does this make to your quality of life or your day to day living?

**1.2**

- Does your nurse provide education to you about Parkinson’s?
- Could you share any examples?
- What difference has this education made to you?
- Where else do you get education from?
- What difference has this made?

**1.3**

- What is the difference between ‘education’ and ‘lifestyle advice’?
- Do you have any examples you could share of when a nurse has shared lifestyle advice with you and what difference this has made?
- Is there a best time or way for you to hear/receive this?

**1.4**

- Does your Parkinson’s nurse include the friends, family, or carer(s) that supports you through your Parkinson’s journey?
- How does the nurse support them?
- What difference does it make?

**Section 2- Access**

Explain:

*We are wondering how different elements of increased access to specialist services works and what impact this has. The following questions are specifically related to how you feel this works in practice for you and how you feel about access.*

**2.1**

- In some cases, Parkinson’s nurses make referrals to Parkinson’s UK, social services, exercise groups, or other NHS services. Is this your experience?
- If you were referred to other clinics and services did you follow through with the referral and what happened?

**2.2**

- How do you contact your Parkinson’s nurse?
- Is it easy to get hold of them?
- What would be the ideal way of accessing your nurse?
- Do you travel to appointments? How do you do this? Can you tell me about who comes with you?

**2.3**

- Would you say your Parkinson’s Nurse is your key ‘go to’ person for your care?
- What would you be likely to contact them for? And what would you choose to somewhere else for?
- Do you think having a go to person reduces the number of different contacts and appointments you need to make?

**Section 3- Medication and Monitoring**

Explain:

*We are wondering how medication and monitoring works across difference service models and what the impact of these are on PwP. The following questions are specifically related to how you feel this works in practice for you and how you feel about medication, prescription, and monitoring.*

**3.1**

- Does your Parkinson’s nurse issue you with prescriptions? (consultant/GP)
- Could you tell me how you get your medications?
- Is this the best way for you? (what’s ideal and why?)
- Do you understand all the medications you are on and why?
- Does your nurse discuss medication with you? (rationale/side effects)
- Do you feel like you work in partnership with your nurse to manage your Parkinson’s? How so?
- Do you always follow your nurse’s advice? (why?)
- Do you get advice from anywhere else on medication? Has this made you make any changes to what you take and when?

**3.2**

- Do you have regular planned appointments? What and where are these?
- Do you often have to ask for additional help or troubleshoot problems around these times? Can you tell me any examples?
- Do you think it is possible that you could be offered ‘too much’ support from a Parkinson’s nurse? Is there such a thing as ‘too-much’? Could you explain why you think this?

**3.3**

- Can you tell me about your Parkinson’s journey with a nurse?
- Would you say having a nurse has made more of a difference at sometimes than others? Could you share an example?

**Step 3: Closing**

**“We’ve completed all the planned questions.** **Is there anything you would like to add before we finish?**

Thank you very much for your contribution. Please feel free to contact me again if there is anything more, I can share about the study.

**Would you like to receive a summary of the outcomes of the study?”**

State time/date of recording.

**CP Interview Schedule (No Nurse)**

**Commence recording as soon as you begin the interview** – including the preliminary discussion.

**Step1: Introduction**

“Good Morning/Afternoon/Evening. **Thank you for agreeing to be interviewed. My name is Katy McEwan. I** work at Northumbria University and am one of the researchers on the USP study.

The purpose of this study is to explore scope and value of the Parkinson’s Specialist Nurse.”

“**Please could I check that you have read and understand the information sheet?** Just to reassure you that everything you say is confidential and you will not be named in any report/paper to come out of the study. Only the research team and transcriber will have access to the recording, which will be destroyed once transcribed. It’s likely that the interview will take between 45 and 60 minutes but please let me know if you’d like to stop at any time for a break or if you would prefer to stop the interview completely. And to reiterate, you are free to withdraw from the study at any time without giving a reason.”

Please obtain verbal consent to participate- **“Can I confirm that you consent to take part in the interview and for the interview to be recorded?”**

“To help you understand what to expect – there are a couple of introductory questions then following questions come under three different themes– I will let you know when we move between sections, so you are aware of the pace and where we are in the interview.”

“**Have you any questions before we start**?”

**Step 2: Interview**

**General**

- We know that Parkinson’s supporters like to be called different things (carer/pillar/family/friend) can I ask what you like to be called and why?
- We will talk about this in different ways in the upcoming questions, but generally, how do you feel about services for people with Parkinson’s?

**Section One**

Explain:

*We are wondering how different aspects of the counselling element in the relationship between the PwP and the nurse works and how it impacts on quality of life and interaction with other services. The first section of questions is specifically related to how you feel that might work in practice.*

**1.1/1.4**

- Where do you both receive emotional support from?
- What difference does this make to your quality of life and day to day living?
- Could you describe what difference you think the emotional support of a nurse would make?
- Who else supports you both through your Parkinson’s journey? Could you share any examples?

**1.2**

- Where do you receive education about Parkinson’s from?
- Could you share any examples of what you have learnt and the difference it has made to you?
- Would it make a difference if you could access education from a Parkinson’s nurse? How so?

**1.3**

- What is the difference between ‘education’ and ‘lifestyle advice’?
- Where do you get ‘lifestyle advice’ from and what difference has this made?
- Would you expect to get lifestyle advice from a Parkinson’s nurse?

**Section 2- Access**

Explain:

*We are wondering how different elements of increased access to specialist services works and what impact this has. The following questions are specifically related to how you feel this works in practice for you and how you feel about access.*

**2.1**

- What services does the pwp you care for access inside or outside of the NHS?
- How did you find out about them?

**2.2**

- How do you contact the different services and providers when you need them?
- What does(n’t) work when you do this?
- Do you support in all appointments? How do you do this? Does anyone else help? What can you not do because you are doing this?

**2.3**

- Do you have a key ‘go to’ person for Parkinson’s care?
- What would you/the pwp be likely to contact them for? And what would you/the pwp choose to go somewhere else for?
- Does having a ‘go to’ person reduce the number of different contacts and appointments you need to make? Can you think of an example?

**Section 3- Medication and Monitoring**

Explain:

*We are wondering how medication and monitoring works across difference service models and what the impact of these are on PwP. The following questions are specifically related to how you feel this works in practice for you and how you feel about medication, prescription, and monitoring.*

**3.1**

- Who do you talk to about Parkinson’s medication?
- Who discusses medication changes with you- why changes need to be made and what that will mean?
- Do you/the pwp understand all the medications they are on and why?
- Does the pwp always take their medication as prescribed? Could you explain why?
- Who issues prescriptions to the pwp?
- How does the pwp get their medications? (do you pick them up? From where? How often? Do you ensure timings/dose? How so?)
- Is this the best way for you? (what is ideal and why?)

**3.2**

- Who currently monitors the Parkinson’s of the person you care for?
- How often do they see them?
- Do you/they have to ask for additional help or troubleshoot problems with anyone? Can you share any examples of what that means for you?
- Do you think it is possible that you/the pwp could be offered ‘too much’ support from anyone? Is there such a thing as ‘too-much’? Could you explain why you think this?

**3.3**

- Can you tell me about the Parkinson’s journey so far and when you have each needed the most support?
- Who has given you support and care at different times and what has this meant to you?

**Step 3: Closing**

**“We’ve completed all the planned questions.** **Is there anything you would like to add before we finish?**

Thank you very much for your contribution. Please feel free to contact me again if there is anything more, I can share about the study.

**Would you like to receive a summary of the outcomes of the study?”**

State time/date for the recording.

**CP Interview Schedule (Nurse)**

**Commence recording as soon as you begin the interview** – including the preliminary discussion.

**Step1: Introduction**

“Good Morning/Afternoon/Evening. **Thank you for agreeing to be interviewed. My name is Katy McEwan. I** work at Northumbria University and am one of the researchers on the USP study.

The purpose of this study is to explore scope and value of the Parkinson’s Specialist Nurse.”

“**Please could I check that you have read and understand the information sheet?** Just to reassure you that everything you say is confidential and you will not be named in any report/paper to come out of the study. Only the research team and transcriber will have access to the recording, which will be destroyed once transcribed. It’s likely that the interview will take between 45 and 60 minutes but please let me know if you’d like to stop at any time for a break or if you would prefer to stop the interview completely. And to reiterate, you are free to withdraw from the study at any time without giving a reason.”

Please obtain verbal consent to participate- **“Can I confirm that you consent to take part in the interview and for the interview to be recorded?”**

“To help you understand what to expect – there are a couple of introductory questions then following questions come under three different themes– I will let you know when we move between sections, so you are aware of the pace and where we are in the interview.”

“**Have you any questions before we start**?”

**Step2: Interview**

**General**

- We know that Parkinson’s supporters like to be called different things (carer/pillar/family/friend) can I ask what you like to be called and why?
- We will talk about this in different ways in the upcoming questions, but generally, how do you feel about services for people with Parkinson’s?

**Section One**

Explain:

*We are wondering how different aspects of the counselling element in the relationship between the PwP, the FFC, and the nurse works and how it impacts on quality of life and interaction with other services. The first section of questions is specifically related to how you feel that might work in practice.*

**1.1 / 1.4**

- Does the Parkinson’s nurse provide the pwp with emotional support? Can you share any examples?
- What difference does this emotional support make to their quality of life or day to day living?
- Does your Parkinson’s nurse provide emotional support to you directly? Can you share any examples of this and what it has meant to you?

**1.2**

- Could you share any examples of when a Parkinson’s nurse has provided education to the pwp you support and the difference this has made?
- Does the nurse provide education to you directly? Can you share any examples?
- Where else do you both get education about Parkinson’s from?
- What difference has this made?

**1.3**

- Do you think ‘education’ and ‘lifestyle advice’ are different things?
- Do you have any examples you could share of when a nurse has shared lifestyle advice with you or the pwp and what difference it made?
- Is there a best time or way for you or the pwp to hear/receive this?

**Section 2- Access**

Explain:

*We are wondering how different elements of increased access to specialist services works and what impact this has. The following questions are specifically related to how you feel this works in practice for you and how you feel about access.*

**2.1**

- In some cases, Parkinson’s nurses make referrals to Parkinson’s UK, social services, exercise groups, or other NHS services. Is this your experience?
- If the pwp was referred to other clinics and services was the referral followed through and what happened?
- Have you been signposted to anything that has helped you?

**2.2**

- How do you/the pwp contact the Parkinson’s nurse?
- Is it easy to get hold of them?
- What would be the ideal way of accessing the nurse?
- Do you attend and/or support all appointments?
- What does this mean to you to do this? Do you have to take time away from anything else? What are the costs to you?

**2.3**

- Would you say your Parkinson’s Nurse is your key ‘go to’ person for the pwp?
- Are they your key ‘go to’ person? (if not, who is?)
- What would you be likely to contact them for? And what would you choose to go somewhere else for?
- Do you think having a go to person reduces the number of different contacts and appointments needed for the pwp?

**Section 3- Medication and Monitoring**

Explain:

*We are wondering how medication and monitoring works across difference service models and what the impact of these are on PwP. The following questions are specifically related to how you feel this works in practice for you and how you feel about medication, prescription, and monitoring.*

**3.1**

- Does your Parkinson’s nurse issue the pwp with prescriptions? (consultant/GP)
- Could you tell me how you get their medications?
- Is this the best way for you both? (what is ideal and why?)
- Do you both understand all the medications they are on and why?
- Does your nurse discuss medication with either of you? (rationale/side effects)
- Do you feel like you work in partnership with your nurse to manage the pwp Parkinson’s? How so?
- Do you both always follow the nurse’s advice? (why?)
- Do you manage the medication at home (dose/timings)?
- Do you get advice from anywhere else on medication? Has this made you make any changes to what they take and when?

**3.2**

- Does the pwp have regular planned appointments? What and where are these?
- Do you often have to ask for additional help or troubleshoot problems around these times? Can you tell me any examples?
- Do you think it is possible that you could be offered ‘too much’ support from a Parkinson’s nurse? Is there such a thing as ‘too-much’? Could you explain why you think this?

**3.3**

- What has having a Parkinson’s nurse meant to you and the pwp over your Parkinson’s journey to date?
- Would you say having a nurse has made more of a difference at sometimes than others? Could you share an example?

**Step 3: Closing**

**“We’ve completed all the planned questions.** **Is there anything you would like to add before we finish?**

Thank you very much for your contribution. Please feel free to contact me again if there is anything more, I can share about the study.

**Would you like to receive a summary of the outcomes of the study?”**

State time/date for the recording.

**Nurses Interview Schedule**

**Please commence recording as soon as you begin the interview – including the preliminary discussion.**

**Step1: Introduction**

“Good Morning/Afternoon/Evening. Thank you for agreeing to be interviewed. My name is…… I am an academic at Northumbria University and one of the researchers on the study.

The purpose of this study is to explore scope and value of the Parkinson’s Specialist Nurse.”

“Please could I check that you have read and understand the information sheet? Just to reassure you that everything you say is confidential and you will not be named in any report/paper to come out of the study. Only the research team and transcriber will have access to the recording, which will be destroyed once transcribed. It’s likely that the interview will take between 30-60 minutes but please let me know if you’d like to stop at any time for a break or if you would prefer to stop the interview completely. And to reiterate, you are free to withdraw from the study at any time without giving a reason.”

Please obtain verbal consent to participate

**“Can I confirm that you consent to take part in the interview and for the interview to be recorded?”**

**“Have you any questions before we start?”**

**There are three sections of questions under different overall themes- and each have sub-sections – I will let you know when we move sections so you are aware of the pace and where we are in the interview.**

**General**

- Can you tell me a little of how you came to work in Parkinson’s nursing?
- Can you tell me about your day to day role?
- How do you feel about services for People with Parkinson’s?

**Section One**

Explain:

*We are wondering how different aspects of the counselling element in the relationship between the PwP and the nurse works and how it impacts on quality of life and interaction with other services. The first section of questions is specifically related to how you feel that might work in practice.*

**1.1**

- We are investigating the value of the emotional support provided to the PwP from the nurse. How do you think the emotional support you provide to PwP and/or FFC helps them in their day-to-day lives? Can you share any examples?
- To what extent is providing emotional support to PwP and/or FFC is an important part of your role? What are the benefits and challenges of this?

**1.2**

- How does the education you provide, through sharing your specialist knowledge with the PwP, impact their self-management? Can you think of examples where this specialist information has made a difference?
- Do you spend much time informally or formally educating other HCP’s? Can you explain the benefits and challenges of this?

**1.3**

- What is ‘lifestyle advice’ and how is it different to ‘education’?
- Can you explain the form and function of lifestyle advice in your day-to-day practice?
- Does the lifestyle advice you give impact outcomes for PwP? Do you have any examples you can share?

**1.4**

- What is your experience of supporting family, friends, and carers through their Parkinson’s journey?
- How do you think this affects outcomes for the PwP?

**Section 2- Access**

Explain:

*We are wondering how different elements of increased access to specialist services works and what impact this has. The following questions are specifically related to how you feel this works in practice for you and how you feel about access.*

**2.1**

- We are interested in the links provided by the nurse to the PwP, the signposting and referrals they do and what the impacts of this are. Can you share any stories of how this works in your practice and what it has meant for you and the PwP?

**2.2**

- How does direct access to a specialist nurse by a PwP work in your service? For example, do you have a telephone advice line or dedicated email?
- What are the benefits and challenges of providing this?
- What is your experience of how patients and carers engaged with these services?
- What are the ideal service settings for access for PwP and nurses?

**2.3**

- What is your opinion of the specialist Parkinson’s nurse as key ‘go to’ contact for the PwP?
- Can you tell me how this has worked (or not) in your service?
- How do you think having a key ‘go to’ Parkinson’s nurse, who provides all information around care, affects the number of appointments for PwP and their need to contact their GP?

**Section 3- Medication and Monitoring**

Explain:

*We are wondering how medication and monitoring works across difference service models and what the impact of these are on PwP. The following questions are specifically related to how you feel this works in practice for you and how you feel about medication, prescription, and monitoring.*

**3.1**

- Suppose the Parkinson’s nurse prescribes and/or titrates medication for PwP- what are the benefits and challenges of that for patients and nurses?
- If I introduce to you three areas where we think medication management by a Parkinson’s Nurse Specialist may be useful from the survey results could you tell me your thoughts on each with examples of the impact on patients and nurses?
- Adherence
- Speed of treatment
- Nurse knowledge- medication & patient

**3.2**

- How does planned monitoring work in your service?
- What kind of monitoring have you found works best?
- Do you have any examples of monitoring influencing a patient’s need to troubleshoot?

**3.3**

- Do you provide hospital comprehensive discharge plans?
- What difference have you found them to make with PwP?

**3.4**

- In your experience what role does a Parkinson’s nurse play in supporting the PwP to transition between the stages of the condition?
- How does this impact on the PwP? Is your role more critical at some stages of transition than others? Do some stages require more support than others? Do you have any experience or knowledge of how this works you could share? Have you had any specific training which has helped in this?

**3.4**

- Do you work as part of an MPT?
- Can you share which other professionals you work with and what this means for your own practice and for patient care? (e.g. we wonder - possible less need for GP to coordinate care and/or reduced hospital admissions).

**Additional Questions (if have time):**

- What is your experience of participating in CPD?
- What benefits or challenges do you think this brings to nurses and PwP?
- What are your future career plans?
- How do you think engaging with ongoing training/education effects your job satisfaction?

**Step 3: Closing**

**“We’ve completed all the planned questions.** **Is there anything you would like to add before we finish?**

Thank you very much for your contribution. Please feel free to contact me again if there is anything more I can share about the study.

Offer to send a study summary once finished…….

**Would you like to receive a summary of the outcomes of the study?**

Once the interview has completed and the person has ‘hung up’ please state the day, date, and time of interview – so it is recorded – before turning off the recording.
