# Supplementary material for: The impact and value of the Parkinson’s nurse specialist to people with Parkinson’s and their care partners: a grounded theory qualitative study
Source: BMC Nurs. 2024 Oct 28;23:791. doi: 10.1186/s12912-024-02441-7 (PMC11520507; doi:10.1186/s12912-024-02441-7)
Supplement: Supplementary file 6 — Supplementary Material 6 [file 12912_2024_2441_MOESM6_ESM.docx]

**The Value and Impact of the PNS Study**

**Full Final Recommendations:**

1. PNS must be available and accessible to PwP and their CP where required to provide specialist expert emotional, education, and lifestyle advice about this complex condition.

2. Every PwP should have access to a PNS from diagnosis.

3. PNS require the opportunity to build knowledge and communication skills in line with competencies to deliver expert support to PwP and CP.

4. PNS provide significant referral and signposting to PwP which has a positive impact on their health and well-being. They require the opportunity to build these networks and the time to provide this conduit of care to PwP and CP.

5. PNS use their specialist knowledge to provide informal and formal education to other Health and Social Care professionals (HSCP) which improves patient care. Time and resources need to be made available to PNS to continue this.

6. High caseloads and low levels of administrative support impede PNS to provide the person-centred continuity of care for PwP and CP to ensure positive outcomes.

7. PNS must be made available to PwP and CP to provide person-centred continuity of care across the stages of Parkinson’s.

8. PNS must be afforded the time to build relationships and partnerships with PwP and CP through continuity of care to encourage the sharing of pertinent information.

9. Appropriate job planning, and succession planning is required to protect the availability of PNS to PwP and CP.

10. PNS should work towards Non-Medical Prescriber (NMP) status early in their careers to build the confidence and extensive knowledge base required for this useful but complicated role. Pharmacological management and review should be incorporated into specialist nurse training and CPD.

11. PwP should be referred into PNS service at diagnosis and be supported to have regular appointments throughout the stages of their condition so PNS can offer on-going personalised specialist pharmacological support and management.

12. To improve PwP concordance with medication regimens, PNS require time (in caseloads and regional availability) to build reciprocal trusting relationships with PwP.

13. PNS must be accessible and available to support medication management to PwP in acute care to ensure they get their treatment regimens are not disrupted.

14. PNS should be a qualified NMP to ensure speed of treatment to PwP to improve health and well-being outcomes.

15. PNS require appropriate time to be included in caseload to manage medications given this is a task comes with a high administration burden to ensure patient safety.

16. PNS are recognised specialists but boundaries between medical and non-medical prescribers can become blurred and tense if not managed. Workplace policies and procedures should ensure the PNS role is clear and well-supported.
